# Programmable Multi‐Axially Aligned Aerogels via Sequential Freeze‐Casting for Tailored Anisotropy and Tunable Mechanics

**DOI:** 10.1002/advs.76283

**Published:** 2026-06-23

**Authors:** Kiho Sung, Sungchul Shin

**Affiliations:** ^1^ Department of Agriculture Forestry and Bioresources Seoul National University Seoul Republic of Korea; ^2^ Research Institute of Agriculture and Life Sciences Seoul National University Seoul Republic of Korea

**Keywords:** aerogels, bio‐inspired materials, freeze‐casting, mechanical anisotropy, multi‐axial alignment

## Abstract

Biological materials achieve exceptional mechanical resilience and multifunctionality through a hierarchical, multi‐axial alignment, a structural complexity that remains challenging to replicate in synthetic porous materials. Conventional freeze‐casting, while promising for mimicking aligned structures, is typically constrained to unidirectional anisotropy when governed by a single temperature gradient, resulting in poor transverse mechanical stability. Here, we present the Sequential Hybridization by Infiltration and Freeze‐casting Technique (SHIFT), a platform that temporally separates primary scaffold formation and secondary architecture formation to enable the programmable construction of multi‐axial aerogel architectures. By utilizing the primary structure as a geometric template during the secondary freeze‐casting of an infiltrated precursor solution, SHIFT allows for controlled tuning of the secondary alignment angle (0°–90°) and spatial density. Mechanical analysis reveals that orthogonal alignment significantly suppresses mechanical anisotropy, achieving an anisotropy ratio of 2.87, closely matching that of natural cuttlebone (2.89), whereas programming the secondary alignment at oblique angles (30°–60°) enhances cyclic recovery. Furthermore, by integrating radial and vertical freezing strategies, multidirectional volumetric mass transport is achieved. This approach offers a versatile strategy to reduce the dependence of structural formation on a single freezing direction, unlocking new possibilities for engineering porous materials with programmable mechanical and transport properties.

## Graphical abstract

Conventional single‐gradient freeze‐casting typically produces unidirectional porous architectures with limited transverse connectivity. The Sequential Hybridization by Infiltration and Freeze‐casting Technique (SHIFT) addresses this constraint by integrating secondary aligned structures within a preformed primary scaffold. This template‐guided strategy enables bio‐inspired programmable multi‐axial aerogels with reduced mechanical anisotropy, tunable cyclic recovery, and multidirectional fluid transport.

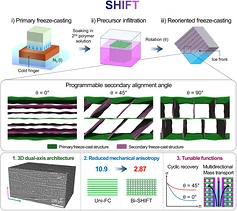

## 1 Introduction

Biological materials adopt complex hierarchical multi‐axial alignment as a core design principle to maintain structural integrity under complex stress environments [1, 2, 3]. To efficiently withstand non‐uniform loads applied from multiple angles, such as hydrostatic pressure, bending moments, and torsion, organisms have optimized their mechanical performance by precisely controlling the alignment angles of building blocks or by interlocking independent alignments within the microstructure [4, 5]. Specifically, osteons in cortical bone effectively disperse tensile and torsional stresses from various directions by adopting a plywood‐like structure where collagen fibers are stacked while rotating at specific angles in each lamellar layer [2, 6]. Similarly, the exoskeletons of crustaceans, such as lobsters and mantis shrimps, form a Bouligand structure in which fiber layers rotate continuously with gradual angle differences. This architecture maximizes fracture energy by continuously twisting the crack path when it propagates in the thickness direction [1, 7, 8]. Such angle control strategies enable the integrated performance of competing functions, such as load bearing and energy absorption, by alleviating stress concentration and multidimensionally deflecting a crack propagation path [1, 7]. In addition to such continuous angle control, nature also achieves microstructural multi‐axial alignment by aligning domains perpendicularly within a single architecture [5]. A representative example is a cuttlebone, which possesses a unique microstructure where horizontal septa and vertical walls intersect in a highly orthogonal manner [9, 10, 11]. In this wall‐septa structure, the septa bounded by the vertical walls function as a transverse support, effectively suppressing local buckling and ensuring structural robustness. Wood also forms a hierarchical orthogonal architecture where vertical vessels and transverse rays connecting them are combined [2, 12, 13]. This interconnected vascular network enables efficient point‐to‐volume fluid transport, allowing rapid vertical wicking while simultaneously distributing nutrients horizontally through radial rays [2, 12, 14]. As such, nature integrates high mechanical strength and multifunctionality within a single architecture through the precise control of angles and the coexistence of independent alignments within the microstructure. Therefore, reproducing these natural multi‐axial alignment principles remains a key challenge in designing next‐generation porous materials, requiring a technological platform capable of programming alignment angles and microstructural complexity within artificial systems.

Freeze‐casting is a process that forms polymer or particle structures by aligning them through the controlled growth of ice crystals along a temperature gradient. It has been regarded as a promising technique for mimicking the aligned porous structures of biological materials [15, 16, 17]. During solidification, polymeric building blocks are excluded from the advancing ice front, concentrating into the inter‐crystalline spaces to form a continuous structure. The subsequent sublimation of these ice crystals leaves behind the unidirectionally aligned porous aerogel. Driven by a directional temperature gradient, this highly anisotropic architecture imparts mechanical characteristics superior to those of conventional isotropic materials [15, 16, 18]. Leveraging this structural tunability, extensive attempts have been made to mimic the architectures of wood, honeycomb, nacre, and bone [1, 2, 15, 18]. For instance, polymer‐based biomimetic research has successfully replicated the brick‐and‐mortar structure of natural nacre using various biopolymers such as silk fibroin [19]. The silk‐based nacre exhibits a 1.67‐fold increase in flexural strength and a 1.37‐fold increase in modulus compared to bulk silk plates. Additionally, bioinspired polymeric woods have achieved a compressive strength of up to 45 MPa, attributed to wood‐like micropore structures [12]. These architectures precisely mimic the hierarchical honeycomb structure of natural wood through the freeze‐casting and sequential thermocuring of thermosetting resins. This indicates that freeze‐casting is a robust platform technology that facilitates the assembly of nanoscale building blocks into macroscopic high‐performance architectures, rather than merely forming porous networks. However, traditional single‐gradient freeze‐casting typically produces uniaxially aligned architectures because ice crystal growth is primarily governed by the imposed temperature gradient [17, 20]. Consequently, reproducing programmable angle control and crossed microstructures comparable to those observed in nature remains a significant challenge. While such uniaxial structures exhibit excellent load‐bearing capacity along the alignment axis, they are often mechanically unstable under transverse stresses because adjacent pore walls are not effectively connected by lateral load‐transfer pathways, unlike the wall‐septa architecture of cuttlebone [17, 18, 21, 22]. As a result, uniaxial aerogels are prone to delamination or buckling under transverse loading, limiting their ability to reproduce the multidirectional damage tolerance of natural materials.

To address the structural weaknesses induced by uniaxial anisotropy, several strategies have been explored, including thermal‐field engineering and microstructural bridge formation. For example, multi‐directional freeze casting has produced cancellous bone‐inspired porous ceramics with multi‐oriented struts and improved mechanical balance across different loading directions, demonstrating the value of apparatus‐defined temperature‐field design [23]. In such single‐step thermal‐field approaches, the interfaces between distinct orientational domains are formed through the coupled growth and impingement of ice fronts, and the spatial arrangement and integration of these domains are intrinsically linked to the imposed thermal‐field geometry. Other microstructural strategies have focused on reinforcing connectivity and load transfer within aligned freeze‐cast architectures by introducing bridge structures between adjacent lamellae or pore walls [17, 21, 24, 25, 26]. Specifically, modulating slurry viscosity or employing freeze‐thaw cycles facilitates particle entrapment to form structural bridges, thereby enhancing load distribution between aligned structures and improving mechanical stability [27, 28]. However, these approaches often rely on spontaneous entrapment, governed by the competition between thermodynamic repulsion and hydrodynamic drag acting on the suspended particles. Since the drag force is proportional to the slurry viscosity (*F*_drag_​ ∝ *ηv*), an increase in viscosity lowers the critical velocity for entrapment (*v*_cr_​ ∝ 1/*η*), causing particles to be dragged and trapped at the advancing ice front [17, 24, 25]. Consequently, bridge formation is strongly influenced by fluid instability and local freezing conditions, making it challenging to independently program the angle or spatial distribution of the secondary alignment [24, 25]. Alternatively, external force fields, such as magnetic [21, 29, 30], electric [31, 32, 33] or ultrasound fields [34] can actively bias particle alignment across the structure. However, their applicability can be limited by the field responsiveness of the building blocks, the need for specialized equipment, and the possible incorporation of responsive additives that may alter the intrinsic properties of the material [35, 36]. Furthermore, even under strong external fields, kinetic competition can persist between particle alignment and the advancing ice front, which may lead to local structural defects or incomplete continuity of the aligned phase [17, 21]. Despite these advances, a structural gap remains between existing freeze‐cast materials and the programmable multi‐axial architectures found in biological systems. Current approaches can effectively generate aligned or locally bridged structures, but independently programming the orientation, spatial distribution, and density of secondary architectures within a preformed porous network remains challenging.

Here, we propose a process platform, termed Sequential Hybridization by Infiltration and Freeze‐casting Technique (SHIFT), to address the geometric limitations of conventional single‐step freeze‐casting. In SHIFT, a primary aligned aerogel is first prepared by directional freeze‐casting and lyophilization. A secondary precursor solution is then infiltrated into the interconnected pore network of this preformed scaffold, followed by secondary freeze‐casting along a prescribed direction. The preformed primary structure thus serves as a geometric template that defines the confined pore space for secondary solidification. This sequential design reduces the direct kinetic competition between primary wall formation and secondary bridge formation that typically occurs during single‐step solidification. As a result, SHIFT enables controlled tuning of the global intersection angle between the primary and secondary alignment axes over the range of 0° to 90°. The resulting multi‐axially aligned architecture forms interpenetrating load‐transfer pathways across the primary and secondary structural domains. This architecture achieves a mechanical anisotropy ratio comparable to that of natural cuttlebone, indicating balanced load‐bearing responses across orthogonal directions. Furthermore, hierarchical orthogonal channels facilitate multidirectional fluid transport and mitigate the hydraulic anisotropy of unidirectional structures. Our platform provides a technical route for implementing refined multi‐axial designs inspired by biological systems.

## 2 Results and Discussion

### 2.1 Bio‐Inspired Structural Design and Sequential Architectural Programming via SHIFT

**FIGURE 1:**
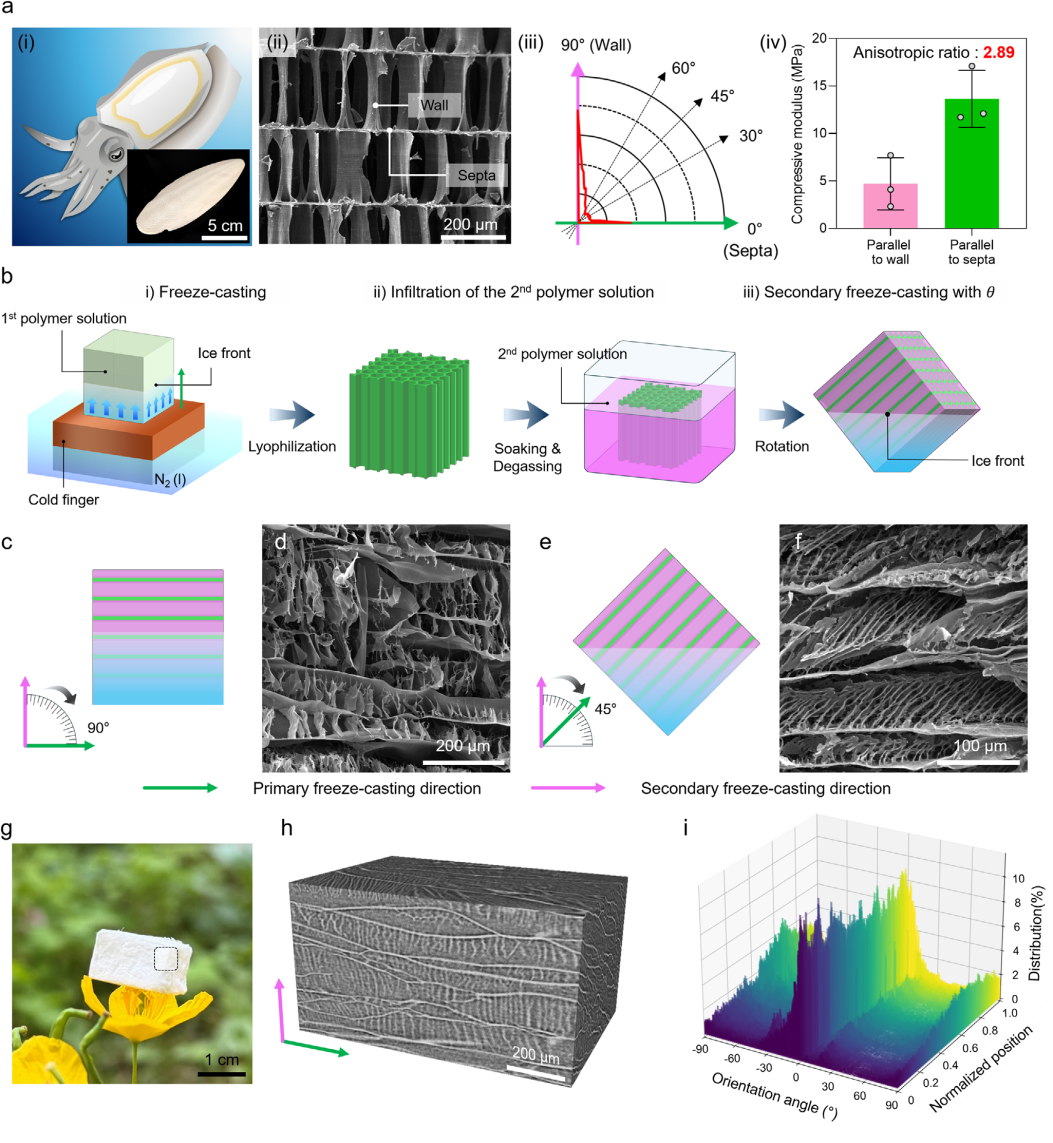
Bio‐inspired architectural design via Sequential Hybridization by Infiltration and Freeze‐casting Technique (SHIFT). (a) Bio‐inspiration derived from the architecture of the cuttlebone. (panel i) Illustration of a cuttlefish and a photograph of a cuttlebone from a cuttlefish (*Sepia officinalis*). (panel ii) Cross‐sectional SEM image showing the natural wall‐septa microstructure. (panel iii) Angular distribution profile of wall and septa, confirming orthogonality. (panel iv) Compressive modulus depending on the compression direction and the anisotropic ratio of the cuttlebone. Data are presented as mean ± SD (n = 3). (b) Schematic illustration of SHIFT. (panel i) Primary freeze‐casting of the primary polymer precursor. (panel ii) Vacuum‐assisted infiltration of the secondary polymer solution into the primary template. (panel iii) Secondary freeze‐casting at a programmed angle (*θ*) relative to the primary axis. (c) Schematic illustration of the orthogonal architecture with a programmed angle of 90°. (d) SEM micrograph of the hybrid architecture with *θ* = 90°. (e) Schematic illustration of the multi‐axial architecture with a programmed angle of 45°. (f) SEM micrograph of the hybrid architecture with *θ* = 45°. (g) Photograph of a freestanding agarose/alginate SHIFT aerogel. (h) Three‐dimensional X‐ray microscopy (XRM) reconstruction of the agarose/alginate SHIFT aerogel fabricated at *θ* = 90°. (i) Orientation distribution profile extracted from reconstructed 3D cross‐sectional images of the *θ* = 90° agarose/alginate SHIFT aerogel.

To define the structural design criteria for mimicking biological load‐bearing systems, we first analyzed the cuttlebone of a cuttlefish (*Sepia officinalis*), a natural orthogonal architecture (Figure 1a, panel i). Analysis of the cross‐sectional architecture via scanning electron microscopy (SEM) reveals that the cuttlebone possesses a periodic architecture, where horizontal septa are interconnected by vertical walls (Figure 1a, panel ii). These horizontal septa act as transverse supports orthogonally interconnected with the vertical walls, creating a mechanically robust wall‐septa structure. As presented in the angular distribution profile derived from the SEM analysis, the septa exhibit a highly anisotropic alignment centered at approximately 90° relative to the vertical walls (Figure 1a, panel iii). This multi‐axial configuration mitigates the mechanical anisotropy characteristic of unidirectional porous architectures. The compressive moduli measured parallel to the septa and walls were 13.6 and 4.7 MPa, respectively, yielding an anisotropy ratio of 2.89 (Figure 1a, panel iv). This structural integration of orthogonal components facilitates effective stress distribution, which significantly enhances the stability under multi‐directional loading conditions by preventing localized failure [37].

To artificially reproduce such intricate multi‐axial alignments based on the orthogonal design principle, we introduced the Sequential Hybridization by Infiltration and Freeze‐casting Technique (SHIFT) (Figure 1b). SHIFT utilizes a sequential protocol for constructing hierarchical architectures through temporally separated freezing steps. First, a polymer precursor solution, represented by 1.5 wt.% agarose, was subjected to freeze‐casting to form a primary aligned structure (Figure 1b, panel i). Following lyophilization, the secondary polymer precursor solution, exemplified by 1.0 wt.% alginate, was infiltrated into the primary structure, which functioned as a structural template. To promote uniform pore filling and interfacial contact, the structure was subjected to vacuum degassing, which helped remove air bubbles trapped within the channels (Figure 1b, panel ii). Subsequently, the infiltrated structure was rotated by a specific angle (*θ*) relative to the primary freezing axis, followed by secondary freeze‐casting (Figure 1b, panel iii). This stepwise approach temporally separates primary scaffold formation from secondary architecture formation. Because the primary aligned scaffold is preformed before secondary solidification, SHIFT reduces the direct kinetic competition between primary wall formation and secondary bridge formation that typically occurs during single‐step solidification. The primary structure maintains its structural integrity and provides a geometric template for the programmed formation of secondary aligned structures within the interstitial spaces during secondary freeze‐casting. The secondary ice crystals propagate along the re‐oriented temperature gradient, enabling the integration of multiple alignment axes within a monolithic architecture. Thus, SHIFT programs the global orientation of the secondary phase through the imposed secondary temperature gradient, while the preformed template defines the confined pore space and transport pathways that influence local secondary‐wall continuity and density during freezing.

To evaluate the programmed control of microstructural alignment achieved via SHIFT, we characterized the synthesized hybrid aerogels using SEM. When the secondary freeze‐casting was programmed at an intersection angle of *θ* = 90°, the microstructure exhibited a distinct ladder‐like architecture, closely resembling the wall‐septa geometry of the natural cuttlebone (Figure 1c,d). SEM imaging reveals that the secondary polymer structure grows perpendicular to the primary template and bridges the parallel channels, while the primary structure largely preserves its orthogonality and structural integrity without gross deformation or collapse during infiltration and secondary freeze‐casting (Figure 1d). To demonstrate the capacity of SHIFT beyond the orthogonal design, we programmed the secondary alignment at a tilted angle of *θ* = 45° by rotating the primary structural template at a 45° offset relative to the vertical temperature gradient (Figure 1e). Following infiltration with the secondary precursor solution, the template was positioned on a cooling source to initiate solidification from the bottom surface. During the secondary freeze‐casting process, ice crystals propagated vertically through the tilted channels, forming a structure in which continuous bridges interconnect the primary template (Figure 1f). At the macroscopic scale, the programmed multi‐axial architecture was fabricated as a freestanding agarose/alginate SHIFT aerogel (Figure 1g). To further examine the three‐dimensional structural continuity, X‐ray microscopy (XRM) analysis was performed on the agarose/alginate aerogel fabricated via SHIFT at *θ* = 90° (Figure 1h). The 3D tomographic reconstruction revealed that the primary aligned network was largely preserved, while secondary structural domains were formed within the interstitial spaces of the pre‐existing channels. Furthermore, quantitative orientation analysis of reconstructed cross‐sectional images showed distinct orientation peaks near 0° and 90° across the analyzed volume, supporting the formation of a dual‐axis architecture (Figure 1i). These results indicate that SHIFT enables programmed hierarchical anisotropy and multi‐axial architecture formation by imposing a secondary freezing direction within a preformed porous template, providing access to structural configurations that are difficult to achieve using conventional single‐step freeze‐casting.

### 2.2 Defining Mechanical and Rheological Criteria for Stable Hierarchical Assembly

**FIGURE 2:**
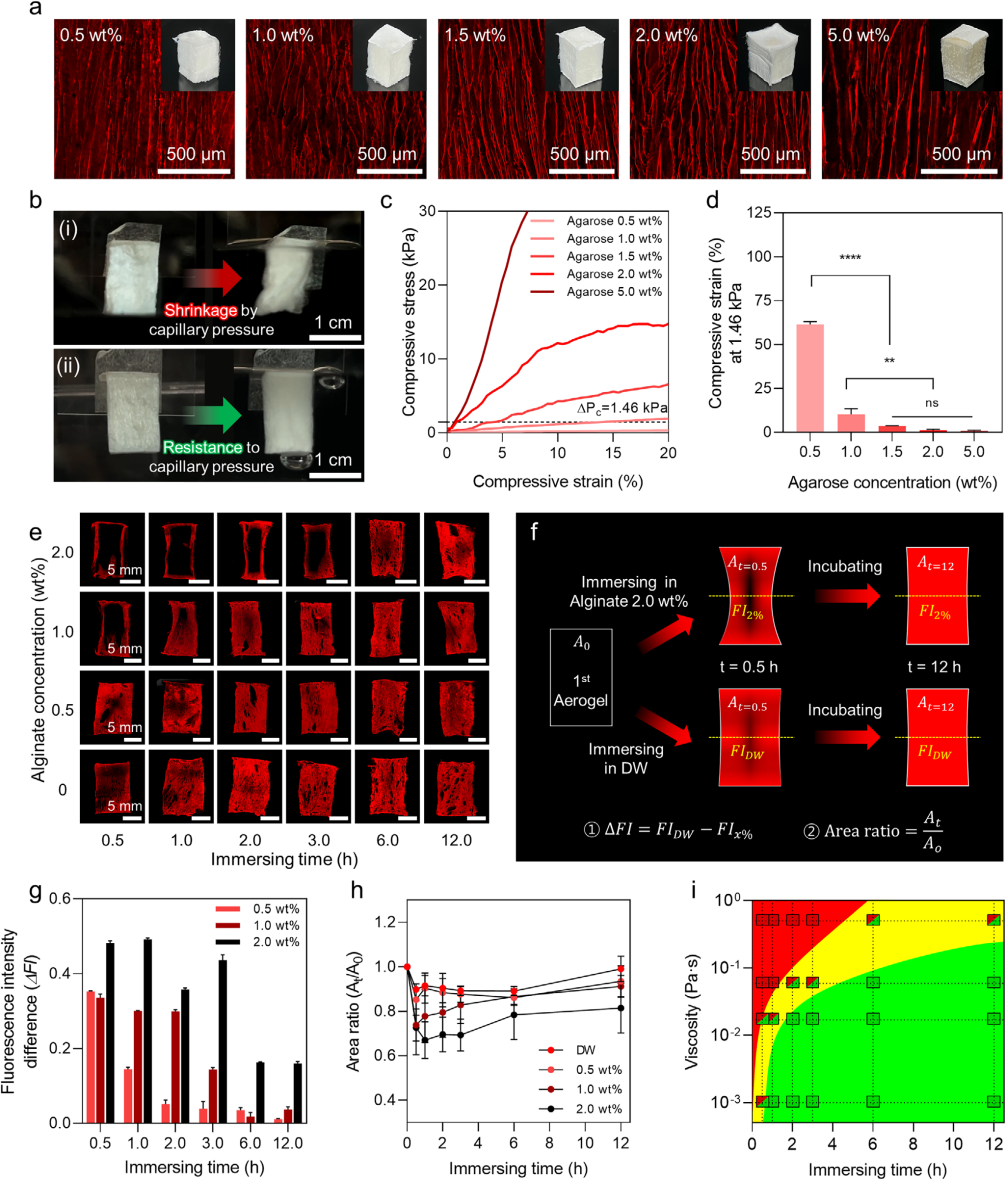
Optimization of primary template stability and secondary infiltration dynamics. (a) Confocal Laser Scanning Microscopy (CLSM) images and photographs (insets) of primary agarose aerogels prepared with concentrations ranging from 0.5 to 5.0 wt.%. (b) Macroscopic observation of structural stability upon immersion: (panel i) structural collapse induced by capillary pressure in 0.5 wt.% agarose aerogel and (panel ii) maintenance of structural integrity in 1.5 wt.% agarose aerogel. (c) Compressive stress–strain curves perpendicular to the alignment axis. The dashed black line represents the estimated capillary pressure (*ΔP*_c_​ ≈ 1.46 kPa) exerted by water. (d) Compressive strain at the critical pressure of 1.46 kPa. (e) Cross‐sectional fluorescence images monitoring the time‐dependent infiltration of alginate solutions with varying concentrations (0–2.0 wt.%) into the agarose aerogel. (f) Schematic definition of quantification metrics: fluorescence intensity difference (*Δ*FI) for infiltration homogeneity and area ratio (*A*_t_​/*A*_0_​) for dimensional stability. (g) Temporal evolution of *Δ*FI, indicating the progression towards uniform infiltration. (h) Time‐dependent variation of the area ratio (*A*_t_​/*A*_0_​), reflecting the swelling and relaxation kinetics during immersion. (i) Processing regime map correlating solution viscosity and immersion time. Green, yellow, and red regions correspond to optimal (stable/uniform), transitional, and unstable (incomplete/collapsed) processing windows, respectively. Data in (d) are presented as mean ± SD (n = 3 independent samples), and data in (g) and (h) are presented as mean ± SD (n = 6 independent samples). Statistical comparisons in (d) were performed using one‐way ANOVA followed by Tukey's post hoc test (ns, not significant; ^**^ *p* < 0.01; ^****^ *p* < 0.0001).

The fabrication of hierarchical architectures via SHIFT requires the primary aerogel template to satisfy two critical criteria. The template is required to maintain sufficient porosity to allow the infiltration of the secondary polymer precursor while simultaneously exhibiting enough mechanical strength to withstand the capillary force generated during the solution infiltration step. These criteria are also important because the preformed template defines the confined pore geometry through which the secondary precursor is transported and subsequently solidified during the secondary freeze‐casting step. To determine the optimal concentration for the primary template, we prepared agarose aerogels with concentrations ranging from 0.5 to 5.0 wt.% (Figure 2a). Microstructural analysis revealed that while the porosity decreased with increasing solid content, the average pore size was similar across all concentrations (Figure S1). This observation indicates that the increase in polymer concentration primarily contributes to the thickening of the primary walls rather than altering the pore dimensions (maintaining an average diameter of ≈ 64 µm), thereby preserving sufficient void space for the unhindered infiltration of the secondary polymer solution. The freezing condition provides an additional processing variable for regulating pore architecture, as a stronger thermal driving force produced finer primary agarose channels and denser secondary alginate structures within the SHIFT architecture (Figure S2). The agarose aerogels, stabilized by temperature‐dependent physical crosslinking [38, 39], exhibited negligible dissolution upon immersion in distilled water as they retained over 96% of their mass after 24 h (Figure S3). Despite the hydrolytic stability, differences in structural stability were observed as a function of agarose concentration (Figure 2b and Movie S1). Following immersion in distilled water, the low‐concentration agarose aerogels underwent severe shrinkage and structural collapse (Figure 2b, panel i), whereas those with higher concentrations (1.5 wt.% and above) retained their original dimensions (Figure 2b, panel ii and Figure S4). This structural degradation is attributed to mechanical failure against capillary pressure rather than chemical dissolution.

To quantify the magnitude of the capillary pressure (*P*_c_​) responsible for the observed structural collapse, the Young‐Laplace equation, expressed as *P*_c_ ​ = 2*γ*cos*θ*/*r*, was applied. Substituting the surface tension of water (*γ* = 72.0 mN·m^−^^1^), the measured contact angle (*θ* ≈ 71°), and the average pore radius (*r* ≈ 32 µm), the capillary pressure was calculated to be approximately 1.46 kPa. This pressure creates a mechanical competition between the capillary force promoting shrinkage and the structural stiffness of the aerogel resisting deformation. This theoretical pressure was compared against the transverse compressive strength of the aerogels, as the capillary forces predominantly induce lateral contraction perpendicular to the aligned pore walls (Figure 2c and Figure S5). Under the calculated capillary load of 1.46 kPa, the 0.5 wt.% aerogel exhibited a compressive strain of ∼61%, indicating mechanical failure beyond its yield point. This severe collapse is further promoted by swelling‐induced wall softening, because water uptake by the hydrophilic agarose walls can simultaneously soften the hydrated walls and generate swelling‐associated internal stress, thereby facilitating local buckling in the thin‐walled structure. This interpretation is consistent with the swelling and re‐drying test, where the 0.5 wt.% agarose aerogel showed severe structural deformation, whereas aerogels with concentrations of 1.5 wt.% and above largely preserved their macroscopic shape (Figure S4). In contrast, the thickened primary walls at higher agarose concentrations significantly enhanced the modulus, resulting in approximately 10% shrinkage for the 1.0 wt.% aerogel. Crucially, aerogels with concentrations of 1.5 wt.% or higher maintained structural stability with negligible deformation (less than 4% strain) under this critical capillary pressure (Figure 2d). This low strain level indicates that the structural response remains within the linear elastic regime, preventing permanent plastic collapse and ensuring that the initial deformation is recoverable. Moreover, the unidirectional alignment formed during the primary freeze‐casting was well‐preserved after immersion for aerogels with agarose concentrations of 1.5 wt.% and above (Figure S6). Based on these results, 1.5 wt.% agarose was selected as the standard concentration to ensure a structural template capable of withstanding multiple freeze‐casting cycles without compromising the primary alignment (Figure S7).

With the intrinsic structural stability of the 1.5 wt.% agarose aerogel confirmed against static capillary forces, the infiltration of the secondary polymer precursor was investigated. Given the high viscosity of the secondary fluid and the rapid pressure equalization required by the vacuum, the aerogel framework experiences substantial mechanical demands during infiltration. Sodium alginate was selected as the secondary fluid to systematically evaluate the effect of viscosity on structural integrity, owing to its rheological dependence on concentration. Rheological measurement quantified this relationship, exhibiting a nonlinear increase in zero‐shear viscosity (*η*_0_​) from 0.02 to 0.5 Pa·s (Figure S8). To visualize the infiltration kinetics under these distinct rheological regimes, the spatial distribution of the secondary alginate was monitored via cross‐sectional fluorescence imaging (Figure 2e). These images indicate that infiltration proceeds as a time‐dependent process, which is hindered by increasing alginate concentration. For the quantitative analysis of this behavior, two specific metrics were defined as the fluorescence intensity difference (*Δ*FI) for infiltration homogeneity and the area ratio (*A*_t_​/*A*_0_​) for dimensional stability (Figure 2f). Monitoring the *Δ*FI quantified the diffusion limitation governed by viscosity. The 0.5 wt.% solution permeated the porous structure, achieving complete saturation (*Δ*FI ≈ 0) within 2 h. In contrast, increasing the concentration to 2.0 wt.% restricted mass transport, resulting in incomplete infiltration even after 12 h due to the increased viscous drag (Figure 2g and Figure S9). Crucially, the 1.0 wt.% solution exhibited a progressive infiltration behavior, reaching equilibrium (*Δ*FI ≈ 0) after approximately 6 h. This trend identifies a viscosity threshold beyond which diffusion‐driven infiltration becomes restricted. We observed that this delayed infiltration is closely correlated with structural deformation induced by the vacuum process. The aerogels exhibited a temporary contraction immediately after immersion, followed by recovery toward equilibrium (Figure 2h). While systems with low‐viscosity solution (0.5 wt.%) recovered to their original volume (*A*_t_​/*A*_0_​ ≈ 1.0) within 30 min, the high‐viscosity solution (2.0 wt.%) induced a pronounced initial contraction to *A*_t_​/*A*_0_ ​≈ 0.60–0.70 and stabilized at a lower equilibrium ratio of ∼0.80 even after 12 h of immersion. For the 1.0 wt.% condition, although an initial contraction occurred, the structure progressively recovered within 6 h. This phenomenon arises because a high‐viscosity solution retards fluid ingress, failing to rapidly equilibrate the internal vacuum pressure. Consequently, this prolonged imbalance in pressure intensifies structural shrinkage and delays volume recovery. Based on this interplay between viscous drag and pressure equilibration, a processing map was constructed to define the operational window for successful infiltration (Figure 2i). This map delineates three distinct operational domains where the red region corresponds to a failure zone with insufficient infiltration and irreversible collapse. The yellow region represents a transient zone of partial infiltration, while the green region denotes a stable processing window achieving uniform infiltration and full dimensional recovery. High‐viscosity solutions fail to equilibrate the internal vacuum pressure, whereas moderate‐viscosity solutions (∼ 0.06 Pa·s) balance infiltration kinetics with mechanical stability. Consequently, 1.0 wt.% alginate was selected as the standard condition for fabricating multi‐axial hybrid aerogels.

### 2.3 Interfacial Affinity as a Determinant for Microscopic Structural Integrity

**FIGURE 3:**
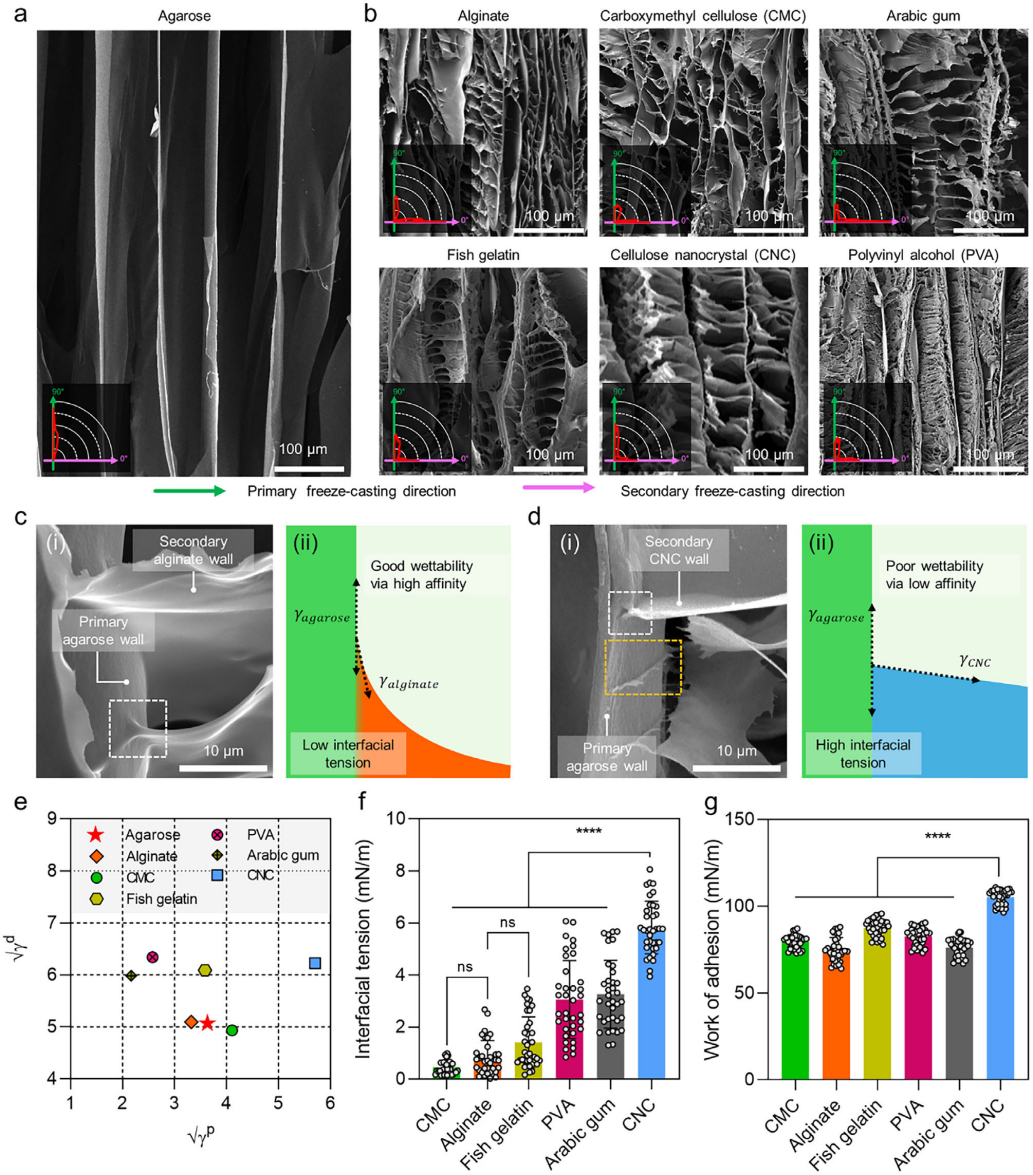
Material versatility and physicochemical analysis of interfacial integration in SHIFT aerogels. (a) Cross‐sectional SEM micrograph of the primary agarose template. (b) SEM images of SHIFT aerogels fabricated using diverse secondary polymers (alginate, fish gelatin, CMC, CNC, arabic gum, and PVA) under normalized viscosity conditions. Insets display orientation distribution profiles confirming orthogonal alignment domains. (c) High‐magnification SEM micrograph (panel i) and corresponding schematic illustration (panel ii) of the agarose‐alginate interface. The distinct concave meniscus (white box) indicates spontaneous wetting and fillet formation. (d) High‐magnification SEM micrograph (panel i) and schematic illustration (panel ii) of the agarose‐CNC interface. The interface displays an abrupt discontinuity and physical gaps. The yellow box highlights polymer residues, providing evidence of localized adhesion despite macroscopic segregation. (e) Surface energy compatibility map plotting the polar (*γ* ^p^) versus dispersive (*γ* ^d^) components derived via OWRK analysis. The radial distance from the agarose reference (red star) indicates the degree of physicochemical affinity. Calculated values of (f) interfacial tension (*γ*_12_​) and (g) work of adhesion (*W*_ad_) for each polymer pair. Alginate and CMC exhibit low *γ*_12_​, whereas CNC displays high *γ*_12_​ despite a high *W*_ad_​. Data in (e) are presented as mean values (n = 6). Quantitative data in (f) and (g) are presented as mean ± SD (n = 36 calculated values). Statistical comparisons in (f) and (g) were performed using one‐way ANOVA followed by Tukey's post hoc test (ns, not significant; ^****^ *p* < 0.0001).

To demonstrate the broad versatility of SHIFT, the range of secondary polymers was expanded to include diverse natural biopolymers (alginate, fish gelatin, carboxymethyl cellulose (CMC), arabic gum, cellulose nanocrystal (CNC)) and a synthetic polymer (polyvinyl alcohol (PVA)). Based on the infiltration dynamics established in the alginate system, the viscosity of all polymer solutions was adjusted to be comparable to that of the 1.0 wt.% alginate solution (Figure S10). Prior to the infiltration process, SEM analysis confirmed that the primary agarose aerogel possessed a well‐defined microstructure of parallel walls aligned along the freezing direction (Figure 3a). This anisotropic porous structure served as the structural template for subsequent hybridization. Following vacuum‐assisted infiltration and secondary freeze‐casting, all polymer systems successfully generated well‐defined porous structures with global alignment perpendicular to the primary axis. The orientation distribution analysis confirms that distinct alignment domains were consistently formed at 0° and 90°, indicating that SHIFT is broadly applicable across the tested secondary polymer systems (Figure 3b, inset). Although the global orientation was universally controlled, the spatial density of the secondary structure varied due to the distinct polymer concentration required to normalize the solution viscosity (∼0.06 Pa·s). For instance, PVA can achieve high concentration while remaining below the target viscosity threshold, which leads to the formation of denser walls due to the higher solid loading. In contrast, CNC exhibits a sharp increase in viscosity even at low concentration, resulting in the formation of thinner walls with lower structural density. Consequently, while viscosity determines infiltration feasibility, the resulting mass concentration defines the densification of the final hybrid architecture. To assess whether SHIFT is limited to agarose‐based primary templates, we additionally fabricated regenerated chitosan and Ca^2^^+^‐crosslinked alginate scaffolds as representative non‐agarose primary templates (Figure S11). Both scaffolds retained their aligned porous architectures after rehydration and re‐lyophilization and exhibited only limited deformation under the estimated capillary pressure. After secondary CMC infiltration and freeze‐casting, aligned secondary structures were successfully formed within the chitosan and alginate primary networks (Figure S12). These results indicate that SHIFT can be extended to non‐agarose primary templates when they possess interconnected porosity, stability in the secondary precursor solution, and sufficient mechanical integrity to resist capillary‐ and swelling‐induced deformation.

To further investigate the interfacial interactions between the primary agarose template and secondary polymers, high‐magnification SEM analysis focused representatively on the alginate and CNC systems, selected for their distinct interfacial behaviors. In the alginate system, a distinct concave meniscus is observed at the hetero‐interface, forming a fillet geometry that secures a broad contact area and a seamless junction (white box in Figure 3c, panel i). This curved profile is indicative of favorable wettability driven by high affinity, consistent with the seamless structural integration depicted in the schematic (Figure 3c, panel ii). In contrast, the CNC system exhibits an abrupt, planar interface without meniscus formation, resulting in a reduced contact area compared to the alginate system (white box in Figure 3d, panel i). This morphology reflects poor wettability, corresponding to the discrete phase separation illustrated in the schematic (Figure 3d, panel ii). Furthermore, this interfacial discontinuity compromises the structural continuity at the junction. However, magnified examination reveals traces of CNC residues adhering to the agarose surface (yellow box in Figure 3d, panel i). These traces provide physical evidence of localized interfacial adhesion, indicating that partial anchoring was established despite the macroscopic segregation. Consequently, these observations suggest that while viscosity control enables the macroscopic formation of multi‐axial architectures, the microscopic integration quality is governed by distinct material‐specific affinities.

To clarify the physicochemical origin of the observed interfacial morphologies, a surface energy compatibility map was established by plotting the polar (*γ* ^p^) versus dispersive (*γ* ^d^) components of each polymer, derived via the Owens‐Wendt‐Rabel‐Kaelble (OWRK) method (Figure 3e and Figure S13). On this map, the radial distance from agarose, which constitutes the primary template, serves as a direct metric for physicochemical affinity. Alginate is positioned adjacent to the agarose reference, indicating a high similarity in their surface energy characteristics. In contrast, CNC is located at the greatest radial distance, reflecting the largest difference in surface properties relative to the agarose reference, which correlates with the morphological distinctness observed between the seamless fillet and abrupt interfaces (Figure 3c,d). Based on these surface energy profiles, we quantified the interfacial tension (*γ*_12_​) to predict the wetting behavior (Figure 3f). The analysis revealed a distinct hierarchy in wetting capability, where alginate exhibited exceptionally low interfacial tension (< 1.0 mN·m^−^^1^) against agarose. This thermodynamic state provides the driving force for spontaneous spreading, resulting in the fillet geometry observed in the microstructure (Figure 3c). Conversely, CNC displayed the highest interfacial tension (∼6.0 mN·m^−^^1^), creating an energy barrier that prevents meniscus formation, leading to the abrupt interface (Figure 3d). However, the work of adhesion (*W*_ad_ *​*) analysis accounts for the localized CNC residues adhering to the agarose surface (Figure 3g). Despite its poor wetting, CNC exhibited a relatively high work of adhesion, comparable to or surpassing that of polymers with high wetting affinity. This suggests that while high interfacial tension prevents conformal coating, the high *W*_ad_ *​* facilitates localized interfacial adhesion. This creates conflicting interfacial behavior for the CNC system, where the high interfacial tension prevents the initial contact by inhibiting wetting, despite the high work of adhesion favoring strong bonding.

### 2.4 Overcoming Mechanical Anisotropy and Optimizing Damage Tolerance via Orthogonal Architectural Engineering

**FIGURE 4:**
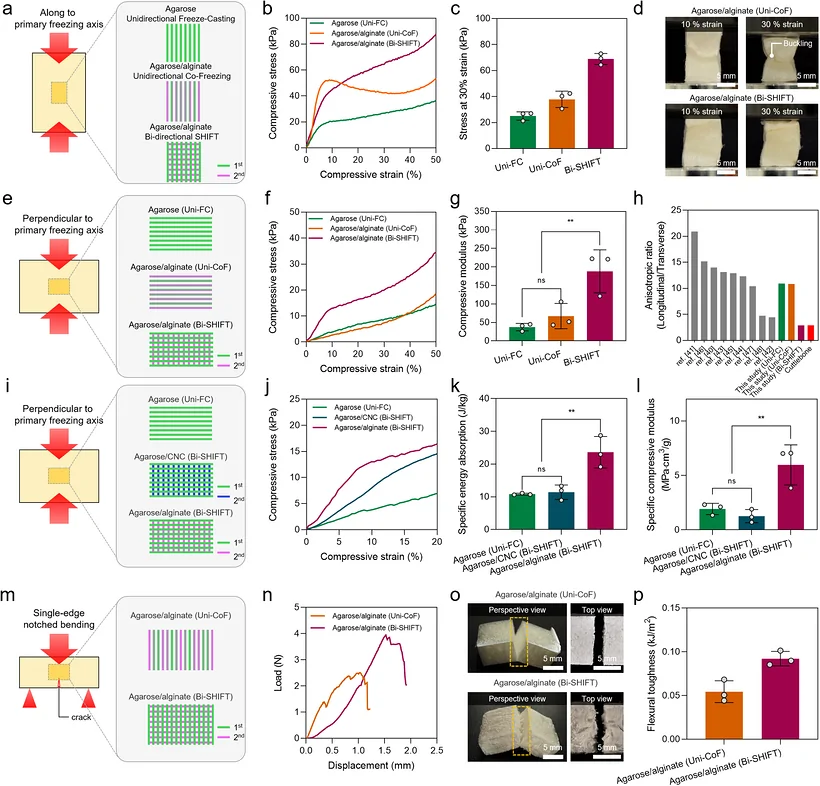
Suppression of mechanical anisotropy and enhancement of damage tolerance via SHIFT architecture. (a) Schematic illustration of the axial compression test along the primary freezing direction. Samples are defined as Uni‐FC (unidirectional freeze‐casting, agarose), Uni‐CoF (unidirectional co‐freezing, agarose/alginate), and Bi‐SHIFT (bi‐directional SHIFT, agarose/alginate). (b) Compressive stress–strain curves of Uni‐FC, Uni‐CoF, and Bi‐SHIFT aerogels. (c) Compressive stress values measured at 30% strain. (d) Photographs showing structural deformation of agarose/alginate aerogels under compression. The top row shows the Uni‐CoF sample undergoing macroscopic buckling (white arrow), and the bottom row shows the Bi‐SHIFT sample at 10% and 30% compressive strain. (e) Schematic illustration of the transverse compression test perpendicular to the primary freezing direction. Samples are identical to those in Figure 4a. (f) Compressive stress–strain curves obtained from transverse compression. (g) Compressive modulus measured in the transverse direction. (h) Anisotropy ratios derived from compressive modulus in the axial and transverse directions, compared with conventional freeze‐cast materials and natural cuttlebone. (i) Schematic illustration of the transverse compression test perpendicular to the primary freezing direction, designed to evaluate the reinforcing effect of interfacial compatibility on mechanical strength. The samples include agarose (Uni‐FC), agarose/CNC (Bi‐SHIFT) with low interfacial affinity, and agarose/alginate (Bi‐SHIFT) with high interfacial affinity. (j) Compressive stress–strain curves obtained from transverse loading. (k) Specific energy absorption calculated as *U*/*ρ*, where *U* is the energy absorbed per unit volume by integrating the stress–strain curve over the 0–10% strain range and ρ is the apparent density. (l) Specific compressive modulus calculated as *E*/*ρ*, where *E* is the compressive modulus derived from the initial linear elastic region, and ρ is the apparent density. The apparent densities used for normalization were 0.0194, 0.0409, and 0.0315 g cm^−^^3^ for agarose Uni‐FC, agarose/CNC Bi‐SHIFT, and agarose/alginate Bi‐SHIFT aerogels, respectively. (m) Schematic illustration of the single‐edge notched bending (SENB) test. (n) Load‐displacement curves obtained from the SENB test. (o) Perspective‐view photographs of Uni‐CoF and Bi‐SHIFT aerogels and microscope analysis of their fracture cross‐sections. (p) Flexural toughness of Uni‐CoF and Bi‐SHIFT aerogels. Quantitative data in (c), (g), (k), (l), and (p) are presented as mean ± SD (n = 3 independent samples). Statistical comparisons in (g), (k), and (l) were performed using one‐way ANOVA followed by Tukey's post hoc test (ns, not significant; ^**^ *p* < 0.01).

To evaluate the structural reinforcement provided by the multi‐axial architecture, we investigated three distinct aerogel types designated as unidirectional freeze‐cast pure agarose (1.5 wt.%, Uni‐FC), unidirectional co‐frozen agarose/alginate (1.5 wt.% / 1.0 wt.%, Uni‐CoF, prepared by freeze‐casting a mixed solution), and bi‐directional SHIFT agarose/alginate (1.5 wt.% / 1.0 wt.%, Bi‐SHIFT) under axial compression (Figure 4a and Figure S14). Generally, freeze‐cast structures exhibit superior mechanical properties when compressed parallel to the freezing direction compared to isotropic aerogels with random pore structure, attributed to the formation of aligned load‐bearing walls. As anticipated, the Uni‐CoF aerogel displayed a higher compressive stress than the non‐freeze‐cast aerogel (Figure S15). To further isolate the structural benefits of SHIFT from simple compositional effects, we focused on the comparison between Uni‐CoF and Bi‐SHIFT. Both hybrid aerogels possess identical material constituents and total solid content, yet differ fundamentally in their pore architecture. While both types of aerogels exhibited increased stiffness compared to the pure agarose‐based Uni‐FC aerogel due to the higher polymer density, a distinct difference in post‐yield deformation behavior was observed between the Uni‐CoF and Bi‐SHIFT aerogels (Figure 4b). The Bi‐SHIFT aerogel maintained enhanced structural integrity at high deformations, exhibiting a compressive stress of 68.88 kPa at 30% strain, which is approximately 1.82‐fold higher than that of the Uni‐CoF (Figure 4c). This mechanical difference is visually evidenced by the distinct macroscopic deformation behavior (Figure 4d and Movie S2). The Uni‐CoF aerogel exhibits pronounced lateral bulging and structural instability beyond 10% strain, indicating the occurrence of macroscopic buckling. In contrast, the Bi‐SHIFT aerogel maintains a stable, uniform shape without pronounced deflection. This observation confirms that the orthogonally aligned secondary walls function as lateral support, effectively reducing the critical buckling length of the primary wall and preventing the premature collapse observed in the unidirectional samples.

Beyond the reinforcement along the primary freezing axis, a critical advantage of SHIFT lies in its ability to mitigate the inherent mechanical anisotropy of freeze‐cast structures. When subjected to compression perpendicular to the primary freezing direction, the Uni‐FC and Uni‐CoF aerogels exhibited immediate structural collapse with minimal load‐bearing capacity regardless of material composition (Figure 4e,f). Notably, their mechanical resistance in this direction was comparable to or lower than that of the non‐freeze‐cast isotropic control, underscoring the inherent structural limitation of unidirectional alignment under stresses perpendicular to the alignment direction (Figure S15). In distinct contrast, the Bi‐SHIFT aerogel demonstrated a robust mechanical response in the transverse direction. Specifically, despite having the same overall polymer content, the Bi‐SHIFT aerogel exhibited a compressive modulus approximately 2.8‐fold higher than that of the Uni‐CoF aerogel (Figure 4g). This enhancement is due to the orthogonal alignment structure formed during the secondary freezing step. The secondary alginate walls function as the primary load‐bearing pillars aligned with the stress axis, while the original agarose structure acts as interconnecting bridges. This architecture effectively extends the load‐transfer pathways across the interfaces, thereby mitigating a key limitation of conventional unidirectional systems, where the lack of such connectivity prevents effective stress transfer. By reinforcing the mechanical strength in both orthogonal directions, the Bi‐SHIFT aerogel exhibited a lower anisotropy ratio than conventional freeze‐cast structures made of various conventional materials including biopolymer (silk [40, 41, 42], lignin‐cellulose composite [43], cellulose [44, 45]) and synthetic polymer (silica composite [46], PEG [47], PVA [48]), serving as a robust bidirectionally reinforced architecture (Figure 4h). Notably, the achieved anisotropy ratio is quantitatively comparable to that of natural cuttlebone, demonstrating the successful emulation of its mechanically balanced architecture. The specific compressive modulus and anisotropy ratio were further compared using Ashby‐type plots to account for density differences among porous materials (Figure S16). The agarose/alginate Bi‐SHIFT aerogel exhibited density‐normalized stiffness values comparable to those of reported natural polymer‐based porous materials, whereas several synthetic polymer‐ and ceramic‐based freeze‐cast systems occupied higher specific stiffness ranges. This comparison clarifies the material‐level boundary of the present agarose/alginate system and emphasizes that SHIFT primarily advances architectural programming for redistributing load‐bearing pathways and reducing mechanical anisotropy.

To further evaluate the critical role of interfacial affinity in determining mechanical efficiency, we analyzed the mechanical performance of the Bi‐SHIFT aerogel with respect to representative secondary material compositions. Agarose/alginate and agarose/CNC Bi‐SHIFT aerogels were selected as representative models possessing distinctly different interfacial morphologies (Figures 3c,d, and 4i). Because these systems required different secondary polymer concentrations under viscosity‐normalized infiltration conditions, the cross‐material mechanical comparison was interpreted using density‐normalized metrics in addition to the stress–strain response. Upon compression, both Bi‐SHIFT aerogels exhibited enhanced stress responses compared with the Uni‐FC control at larger deformation, although the extent and onset of reinforcement differed between the CNC‐ and alginate‐based systems (Figure 4j and Figure S17). However, differences were observed in the density‐normalized energy absorption depending on the secondary material composition of the Bi‐SHIFT aerogels (Figure 4k). The agarose/CNC Bi‐SHIFT aerogel, which has low interfacial affinity with the primary agarose structure, showed no statistically significant difference from the Uni‐FC control. In contrast, the agarose/alginate Bi‐SHIFT aerogel, which has high interfacial affinity with agarose, exhibited significantly higher specific energy absorption. This trend is consistent with the load‐bearing mechanism in the initial deformation regime. While the agarose/alginate aerogel demonstrated immediate structural stiffening from the onset of loading, the agarose/CNC aerogel displayed a delayed onset of structural stiffening. Specifically, in the early deformation region used for energy‐absorption analysis (0–10%), the stress response of the CNC‐reinforced aerogel remained close to that of the pure Uni‐FC aerogel, indicating delayed mechanical engagement of the secondary structure. This macroscopic difference is intrinsically linked to the distinct interfacial morphologies governed by wetting behavior. The agarose/alginate system is characterized by the formation of filleted junctions at the heterojunctions, where low interfacial tension induces a concave meniscus that solidifies into a structural reinforcement with a broad contact area (Figure 3c). This smooth geometric transition effectively minimizes stress concentration and facilitates immediate load transfer between the primary and secondary structures. In contrast, the high interfacial tension in the agarose/CNC system prevents fillet formation, resulting in an abrupt interface with a strictly limited contact area that restricts efficient stress distribution and requires finite deformation to initiate engagement (Figure 3d). Consequently, the alginate‐based system exhibited a higher specific compressive modulus than the CNC‐based system, even though the CNC system contained a higher secondary solid concentration (Figure 4l). These results indicate that the difference in load‐transfer efficiency is not governed by solid loading alone but is strongly associated with the interfacial morphology between the primary and secondary structures.

Biological organisms possessing dual‐axis architectures are often subjected to diverse mechanical stressors beyond simple static compression, requiring robust damage tolerance. To replicate these harsh environmental conditions, we conducted a single‐edge notched bending (SENB) test to investigate the distinct fracture behaviors and crack propagation patterns (Figure 4m). The representative load‐displacement curves reveal distinct improvements in both strength and deformability for the Bi‐SHIFT aerogel (Figure 4n). While the Uni‐CoF aerogel failed at a relatively low peak load with limited displacement, the Bi‐SHIFT aerogel achieved a higher maximum load and sustained structural integrity over a larger displacement range before complete failure. Notably, the Bi‐SHIFT aerogel exhibited a characteristic stepwise fracture behavior during the post‐peak stage. This extended failure process is intrinsically attributed to the crack‐deflecting capability of the secondary structure, as visualized in the fracture path analysis (Figure 4o). While the Uni‐CoF aerogel underwent rapid longitudinal splitting along the primary freezing direction due to the lack of lateral reinforcement, the orthogonal walls in the Bi‐SHIFT structure act as physical barriers that intercept and deflect the propagating cracks. These barriers force the cracks to follow a highly tortuous path, thereby preventing abrupt cleavage. Consequently, this effective energy dissipation mechanism resulted in an enhancement in flexural toughness (approximately 1.8‐fold), confirming the successful engineering of a damage‐tolerant architecture (Figure 4p). Collectively, these results demonstrate that the orthogonally interconnected architecture of the Bi‐SHIFT aerogel provides application‐relevant mechanical advantages not only by increasing axial resistance but also by altering deformation and fracture modes. Compared with the Uni‐CoF aerogel, the Bi‐SHIFT aerogel suppressed post‐yield buckling, enhanced transverse load‐bearing capacity, and promoted crack deflection through a tortuous fracture path. These structural effects resulted in higher stress under large deformation, a higher transverse modulus, and increased flexural toughness. Such architecture‐enabled mechanical responses are potentially relevant to low‐density porous materials that require resistance to compression‐ or bending‐induced collapse, including lightweight protective cores, energy‐absorbing cellular structures, and damage‐tolerant cellular materials.

### 2.5 Deterministic Programming of Mechanical Anisotropy and Fatigue Resilience via Angular Architecture

**FIGURE 5:**
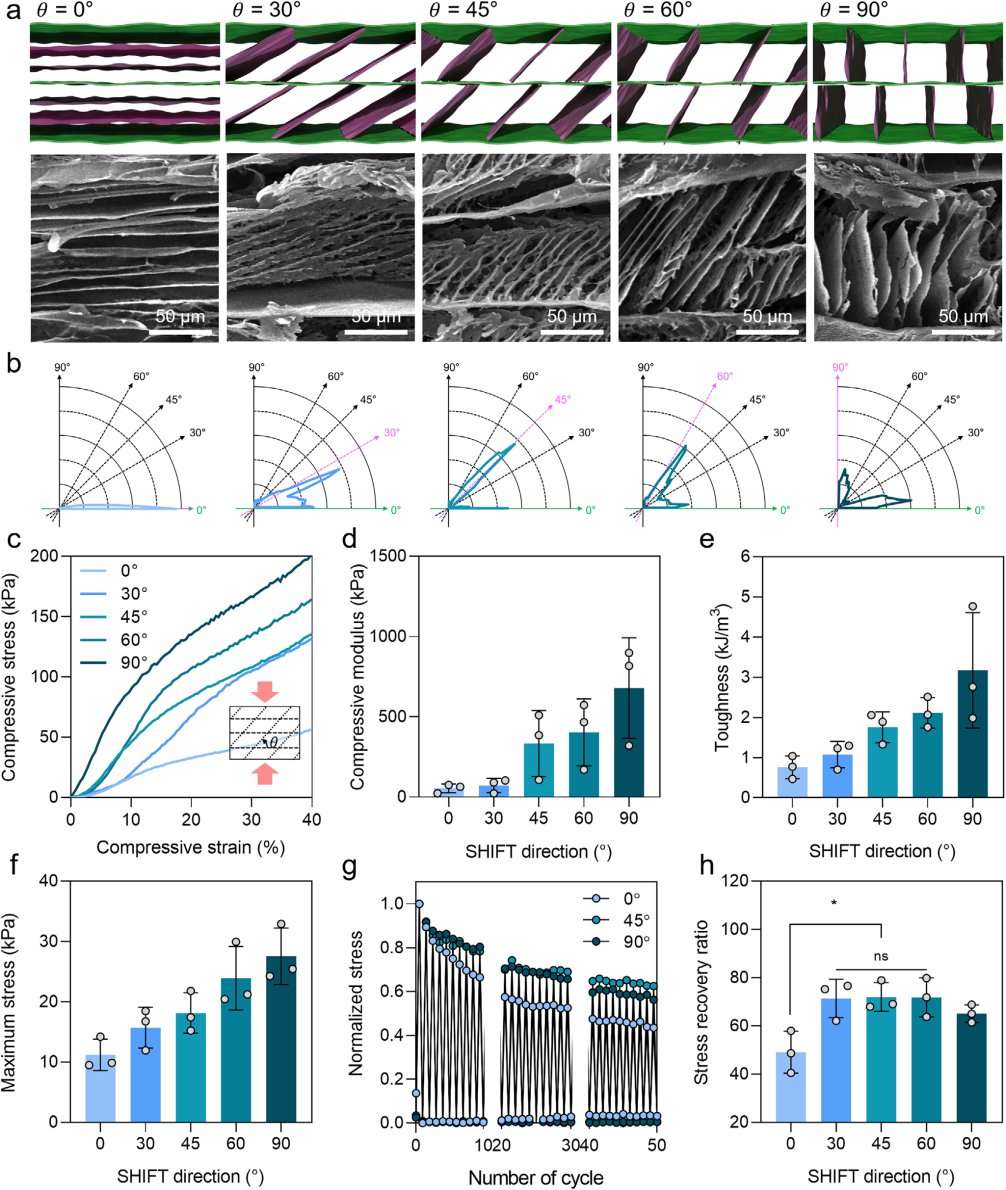
Programmable mechanical anisotropy and cyclic resilience via angular architectural control. (a) Schematic illustrations (top) and corresponding cross‐sectional SEM images (bottom) of SHIFT aerogels engineered with varying secondary freezing angles (*θ* = 0°, 30°, 45°, 60°, and 90°). (b) Orientation distribution profiles quantifying the angular alignment of the secondary structures. (c) Representative compressive stress–strain curves under transverse compression loading perpendicular to the primary freezing axis. (d) Compressive modulus plotted as a function of the secondary freezing angle. (e) Toughness as a function of the secondary freezing angle. (f) Maximum compressive stress measured at varying inclination angles. (g) Evolution of normalized compressive stress during 50 cycles of repeated loading. (h) Stress recovery ratio after 50 cycles of repeated compression. Quantitative data in (d), (e), (f), and (h) are presented as mean ± SD (n = 3 independent samples). Statistical comparisons in (h) were performed using one‐way ANOVA followed by Tukey's post hoc test (ns, not significant; ^*^ *p* < 0.05).

Nature optimizes mechanical performance through specific structural orientations. For instance, the deep‐sea sponge *Euplectella aspergillum* employs diagonal struts (45°) to suppress buckling [49], whereas the intervertebral annulus fibrosus utilizes oblique collagen fibers (30°–60°) to maximize cyclic resilience [50]. To implement this geometric programming strategy, we exploited the versatility of SHIFT to fabricate aerogels with varying secondary freezing angles (*θ*). We controlled the direction of the secondary temperature gradient relative to the primary structural walls to induce secondary wall formation at designated angles of 0°, 30°, 45°, 60°, and 90° (Figure 5a). Orientation analysis quantitatively verified this structural programmability, where the orientation distribution profiles displayed a primary peak near 0° originating from the primary freeze‐casting and distinct secondary peaks shifting precisely with the predefined secondary angles (Figure 5b and Figure S18). This capability represents a significant advance in freeze‐casting, transitioning the technique from simple unidirectional alignment to programmable architectural design, thereby enabling deterministic tuning of mechanical anisotropy.

We next investigated how these distinct architectural configurations translate into macroscopic mechanical performance. Subjected to transverse compression perpendicular to the primary freezing direction, the mechanical response of these architectural variants exhibited a clear dependence on the secondary wall angles (*θ*) (Figure 5c). As the secondary wall angle increased from 0° (parallel) to 90° (orthogonal), the stress–strain behavior shifted from a soft, highly deformable response to a rigid, load‐bearing state. This structural transition demonstrates the precise tunability of mechanical performance, allowing for programmable property modulation across key metrics. First, compressive modulus exhibited a substantial rise as the secondary angle approached 90°, indicating that the increasing alignment of the secondary structures with the transverse loading direction maximizes resistance to initial deformation (Figure 5d). In parallel with this stiffening, the toughness increased steadily as the secondary wall angle increased, suggesting that the 90° architecture dissipates mechanical energy more effectively (Figure 5e). Furthermore, the maximum compressive stress was notably reinforced, reaching its peak at 90° (Figure 5f). This wide‐ranging tunability demonstrates that diverse mechanical properties can be achieved from a single material composition, solely by manipulating the micro‐architecture. This is attributed to the geometric contribution of the secondary walls, which increasingly function as structural pillars aligned with the loading direction as they approach 90°, thereby directly supporting the applied stress.

Beyond static mechanical properties, the angular architecture plays an important role in determining cyclic resilience and fatigue resistance. We evaluated the stress recovery capability of the SHIFT aerogels under 50 cycles of repeated compression (Figure 5g). The alignment angle significantly influenced the stress recovery ratio, revealing a critical trade‐off between stiffness and recoverability. At 0°, the absence of lateral reinforcement resulted in limited load‐transfer pathways, leading to structural collapse and a low recovery ratio. Conversely, increasing the angle to 90° maximized stiffness but resulted in excessive rigidity, which can induce higher energy dissipation and plastic deformation, slightly reducing recoverability compared to the intermediate angle structures (30°–60°) [51]. Notably, the intermediate angles exhibited the highest stress recovery ratios (Figure 5h). Furthermore, a similar angle‐dependent trend was observed over 500 long‐term compressive cycles (Figure S19). The stress recovery ratios after 500 cycles were 29.7% for 0°, 50.2% for 45°, and 41.9% for 90°. The 45° architecture showed the highest average stress recovery among the tested angles, indicating that intermediate‐angle architectures can enhance resistance to long‐term cyclic loading by balancing structural reinforcement and reversible deformation. In these configurations, the inclined secondary walls function as elastic trusses, which effectively distribute stress while allowing for reversible structural flexing. SEM analysis after 50 cycles of repeated compression further supported the angle‐dependent stress‐recovery behavior (Figure S20). The 0° architecture showed compaction and local bending of adjacent aligned layers, whereas the 90° architecture exhibited buckling and local fracture of vertically oriented secondary walls. In contrast, the 45° architecture largely preserved its inclined secondary structures without pronounced fracture, supporting the role of intermediate‐angle walls in distributing stress while allowing reversible deformation. This suggests that, analogous to the oblique collagen fibers in biological tissues, angular alignment within a specific range (30–60°) offers an optimal balance, providing sufficient reinforcement to prevent collapse while maintaining the structural compliance necessary for efficient elastic recovery. This angle‐dependent response defines a design space in which stiffness and cyclic recoverability can be balanced by geometry rather than by changing material composition. The 90° architecture is advantageous when transverse stiffness is prioritized, whereas intermediate‐angle architectures are more suitable for porous systems subjected to repeated deformation because they provide reinforcement while preserving structural compliance. This tunability may be useful for mechanically adaptive porous supports, resilient cushioning structures, and scaffold‐like porous architectures requiring deformation tolerance.

### 2.6 Bio‐Inspired Multidirectional Mass Transport via Hierarchical Radial‐Vertical Integration

**FIGURE 6:**
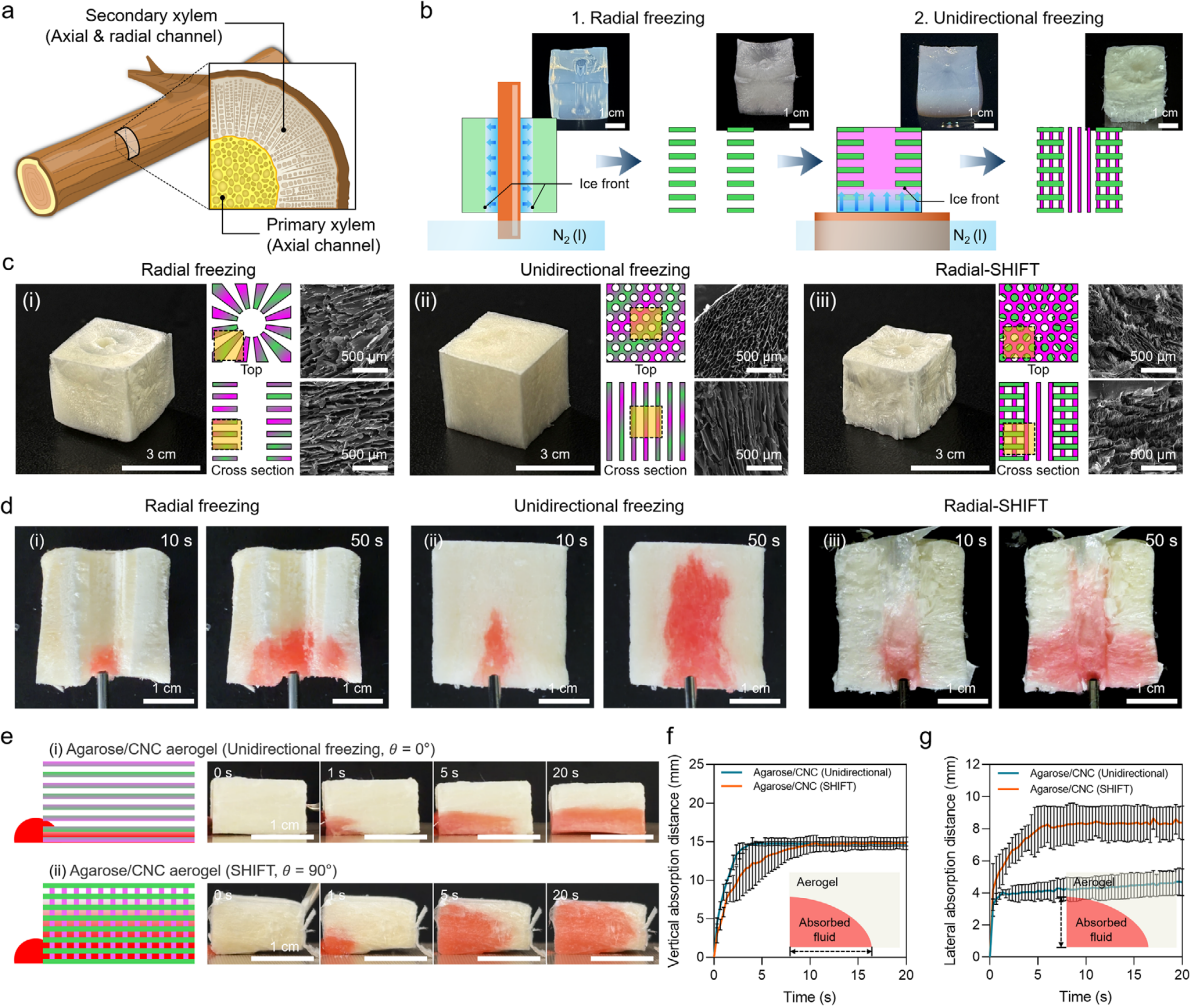
Bio‐inspired multidirectional mass transport via Radial‐SHIFT architecture. (a) Schematic illustration of the hierarchical vascular system in a woody stem, consisting of axial primary xylem and radial secondary xylem. (b) Schematic representation of the Radial‐SHIFT process integrating radial and unidirectional freeze‐casting to fabricate dual‐channel aerogels. (c) Optical photographs (left) and cross‐sectional SEM images (right) comparing the pore architectures of (panel i) radial freezing, (panel ii) unidirectional freezing, and (panel iii) Radial‐SHIFT aerogels. (d) Time‐lapse visualization of dye diffusion in (panel i) radial, (panel ii) unidirectional, and (panel iii) Radial‐SHIFT aerogels, demonstrating macroscopic fluid transport behaviors. (e) Schematic illustrations and corresponding fluid absorption photographs comparing the mass transport capability of (panel i) unidirectional aerogels (*θ* = 0°) and (panel ii) SHIFT aerogels (*θ* = 90°). (f) Vertical fluid absorption distance plotted as a function of time. (g) Lateral fluid absorption distance plotted as a function of time. Data in (f) and (g) are presented as mean ± SD (n = 3).

In biological systems, structural architecture dictates not only mechanical stability but also mass transport efficiency. Nature optimizes this fluid dynamics through hierarchical architectural designs. A prime example is the secondary xylem of a woody stem, which possesses a sophisticated dual‐channel system in which axial vessels transport water vertically and radial rays distribute nutrients horizontally (Figure 6a). This interconnectivity allows trees to achieve efficient point‐to‐volume transport, ensuring that fluid absorbed from the root is rapidly dispersed throughout the entire tissue volume. While freeze‐casting has been widely explored to emulate such porous biological scaffolds, the technique typically generates only unidirectional channels, leading to severe hydraulic anisotropy where fluid flows rapidly in one direction but is obstructed laterally. To recapitulate this biological efficiency, we designed a Radial‐SHIFT that integrates radial and unidirectional freeze‐casting strategies (Figure 6b). We employed cellulose nanocrystals (CNCs) as cellulose‐based secondary building blocks inspired by the cellulose‐rich composition of plant cell walls, utilizing their inherent hydrophilicity to facilitate capillary transport. Additionally, we implemented a citric acid‐mediated thermal crosslinking step after fabrication as a structural stabilization strategy to reduce swelling‐induced collapse of the hydrophilic agarose/CNC network during aqueous transport, thereby helping preserve the pore architecture required for capillary wicking. The fabrication involves a sequential two‐step process in which a radial temperature gradient first forms a horizontally distributed structure, followed by a vertical temperature gradient that induces the growth of axial channels, creating a mutually orthogonal and interpenetrating architecture.

The distinct structural features of the Radial‐SHIFT aerogel are visually confirmed through macroscopic and microscopic comparison (Figure 6c). The radial freezing aerogel (panel i) exhibits a radially aligned channel structure that distributes fluid horizontally in all directions but lacks continuous vertical pathways for bulk transport. Conversely, the unidirectional freezing aerogel (panel ii) possesses vertically aligned channels ideal for upward wicking but completely lacks lateral connectivity, restricting fluid distribution solely to the injection axis. In contrast, the Radial‐SHIFT aerogel (panel iii) successfully integrates both features, displaying a lattice‐like microstructure where vertical channels are periodically intersected by radially aligned structures. SEM analysis confirms that this orthogonal connectivity creates interconnected pores between adjacent walls, establishing continuous 3D flow pathways without compromising the alignment of the primary vertical channels. We validated the functional superiority of this architecture through fluid transport visualization (Figure 6d and Movie S3). When dye solution was injected at the bottom center, the Radial‐SHIFT aerogel demonstrated rapid point‐to‐volume transport, distributing the fluid radially throughout the structure while simultaneously wicking it vertically to the top surface within seconds. This contrasts sharply with the controls, where transport was confined either to the horizontal plane or the vertical axis.

To quantitatively assess the mechanism behind this enhanced multidirectional mass transport, we analyzed the kinetics of lateral fluid diffusion using a simplified orthogonal SHIFT model (Figure 6e). While the unidirectional aerogel allowed rapid vertical transport (*θ* = 0°), it exhibited negligible lateral spreading due to the physical confinement by the parallel walls (Figure 6e, panel i). In contrast, the SHIFT aerogel, with its orthogonal CNC bridges (*θ* = 90°), enabled extensive lateral dispersion (Figure 6e, panel ii). Quantitative tracking revealed that while both systems maintained similar vertical wicking speeds, reaching the saturation height of 15 mm within 20 s (Figure 6f), the SHIFT aerogel achieved a lateral transport distance of 9 mm. This represents a more than twofold increase compared to the 4 mm observed in the unidirectional control (Figure 6g). These results indicate that the secondary CNC structures function as lateral hydraulic bridges that connect otherwise isolated unidirectional channels. Thus, the SHIFT architecture preserves rapid axial wicking while adding transverse redistribution. Such point‐to‐volume transport is particularly relevant to transport‐dominated porous systems in which reactant access, liquid redistribution, and internal surface utilization must be balanced, including catalytic supports, filtration and absorbent media, wick‐based delivery platforms, and fluid‐distribution porous scaffolds.

Because these applications require direct exposure to aqueous fluids, we further evaluated the mechanical response of the Radial‐SHIFT aerogel before and after fluid transport (Figure S21). The aerogel retained its macroscopic integrity after aqueous fluid transport, but its compressive response and modulus decreased substantially under the post‐transport wet condition, indicating that water‐induced softening remains an important design consideration. This result highlights the need to balance hydrophilic transport pathways with wet‐state mechanical stability in future Radial‐SHIFT systems. Although the present agarose/CNC aerogels serve as model architectures, the same design principle could be extended to functional porous materials through the incorporation of catalytic particles, chemically stable polymers, selective interfaces, bioactive components, or conductive phases according to the target application.

The scalability and process complexity of SHIFT should also be considered for practical implementation. In a single directional freeze‐casting step, the lateral dimensions of the primary template can be increased by enlarging the mold and copper plate. However, the final SHIFT architecture requires secondary freeze‐casting after reorienting the preformed primary template. Therefore, the uniformly aligned size of the dual‐axis SHIFT architecture is governed not only by the lateral size of the initial template but also by the maximum stable freezing distance in each sequential freezing direction. In our current fixed cold finger‐based setup, relatively uniform aligned pores were maintained up to approximately 4.5 cm from the cold source, whereas samples exceeding this distance showed broader pore‐size distributions and reduced alignment as the freezing front advanced away from the cold source (Figure S22). This dimensional limitation reflects a general scale‐up challenge in freeze‐casting, where maintaining consistent ice‐growth conditions over long distances from a fixed cold source is difficult. Moving cold‐source or additive freeze‐casting approaches may provide possible routes for future scale‐up by maintaining a shorter and more consistent freezing distance during sequential reoriented freezing [52]. Therefore, further development of large‐area SHIFT architectures will require strategies that preserve directional alignment over larger volumes while enabling controlled secondary freezing after template reorientation. From a process standpoint, SHIFT also involves additional steps compared with single‐step freeze‐casting, including primary freeze‐casting, freeze‐drying, secondary precursor infiltration, angular reorientation, secondary freeze‐casting, and final drying. These additional freezing and drying steps are expected to increase processing time and energy demand relative to single‐step freeze‐casting. A single‐step dual‐gradient protocol may offer a more process‐efficient route for generating globally multi‐oriented pore structures by imposing multiple temperature gradients within one freezing event. However, this simplification would also couple domain formation to a single solidification process, whereas SHIFT enables secondary architectures with prescribed orientation, composition, and interfacial characteristics to be introduced into a preformed porous network. Thus, the additional complexity of SHIFT should be viewed as a trade‐off for sequential architectural integration rather than as a manufacturing‐optimized process at this stage.

## 3 Conclusion

In this study, we established SHIFT as a versatile platform that addresses key geometric constraints imposed by single‐step freeze‐casting, advancing the methodology from simple unidirectional formation to deterministic architectural programming. By temporally separating primary scaffold formation from secondary architecture formation and reducing direct kinetic competition between the two solidification steps, we successfully fabricated complex multi‐axially aligned aerogels that emulate the sophisticated designs of biological systems, including the orthogonal support of cuttlebone, the alternating oblique architectures mimicking the annulus fibrosus and *Euplectella aspergillum* sponge, and the integrated radial and vertical alignment mimicking wood xylem. Our study demonstrates that the macroscopic mechanical and functional properties of porous architectures can be tailored not merely by material composition but through precise geometric control of the microstructural alignment. We established that the introduction of secondary structural domains effectively acts as lateral bracing and elastic trusses, thereby reducing mechanical anisotropy while enabling mechanical robustness and multidirectional mass transport. Furthermore, the broad applicability of SHIFT to diverse polymer systems, governed by solution viscosity and interfacial affinity, highlights its potential as a broadly adaptable processing route for porous materials that satisfy the required infiltration, interfacial, and mechanical stability criteria. This programmable approach provides an architectural design framework for porous materials in which load‐transfer pathways and transport pathways can be engineered with greater independence. With application‐specific material optimization, these principles may be extended to mechanically demanding cellular materials that require buckling resistance, crack deflection, and cyclic recoverability, as well as transport‐dominated porous systems that require axial wicking, lateral redistribution, and point‐to‐volume fluid delivery.

## 4 Experimental Section

### 4.1 Materials

Micro agar powder was purchased from Duchefa Biochemical (Haarlem, Netherlands). Sodium alginate was obtained from Junsei chemical Co., Ltd. (Tokyo, Japan). Polyvinyl alcohol (PVA, DP: 1,500) and citric acid were obtained from Duksan Pure Chemicals (Ansan, Korea). Carboxymethyl cellulose (CMC, Mw: 90,000, degree of substitution: 0.7), gelatin from *cold water fish skin*, high molecular chitosan, Rhodamine B and diiodomethane were purchased from Sigma–Aldrich (St. Louis, MO, USA). Arabic gum was purchased from Fisher Scientific (Waltham, MA, USA), and cellulose nanocrystal (CNC, dimensions: 2–5 nm × 100 nm) was purchased from CelluForce (Montreal, Canada). All chemicals were used as received without further purification. Deionized water was used for the preparation of all aqueous solutions.

### 4.2 Fabrication of SHIFT and Control Aerogels

Aerogels were fabricated using five distinct protocols, including non‐freeze‐cast, unidirectional freeze‐casting (Uni‐FC), unidirectional co‐freezing (Uni‐CoF), radial freeze‐casting, and SHIFT. The polymer concentrations used for each method are listed in Table S1. Except for agarose, all polymer solutions were prepared as 5× stock solutions and diluted to the target concentrations prior to use. To minimize batch‐to‐batch variation in the cooling condition, all cold‐finger‐based directional freeze‐casting steps were conducted using the same mold geometry, sample volume, sample position, cold‐finger configuration, and freezing protocol. Before sample placement, the copper cold finger was pre‐cooled under the same liquid nitrogen cooling condition for a fixed equilibration time. During freezing, the liquid nitrogen level in the reservoir was maintained constant by continuous replenishment to minimize variation in the cold‐finger cooling environment across batches.

For non‐freeze‐cast aerogel (isotropic control), a mixed solution of 1.5 wt.% agarose and 1.0 wt.% alginate was poured into a mold, frozen at ‐80 °C in a deep freezer, and freeze‐dried for 48 h.

For unidirectional freeze‐casting (Uni‐FC), pure agarose solutions were prepared with concentrations ranging from 0.5 to 5.0 wt.% to investigate structural evolution. The solution was poured into a mold (dimensions: 10 mm × 10 mm × 15 mm) and allowed to gel at room temperature. For large‐area Uni‐FC, the solution was poured into a mold (dimensions: 50 mm × 80 mm × 15mm) and allowed to gel at room temperature. The hydrogel was placed on a cold finger cooled by liquid nitrogen (−196 °C) to induce ice crystal growth along a unidirectional temperature gradient. The frozen samples were freeze‐dried for 48 h.

For unidirectional co‐freezing (Uni‐CoF), composite precursor solutions were prepared depending on the specific experimental purpose. For mechanical characterization, a mixed solution of 1.5 wt.% agarose and 1.0 wt.% alginate was employed. For mass transport evaluation, a composite solution comprising 1.5 wt.% agarose, 2.0 wt.% CNC, and 0.2 wt.% citric acid was used. The fabrication protocol followed the same procedure as Uni‐FC, which involved pouring the mixture into a mold, gelling at room temperature, directional freezing on a cold finger (‐196 °C), and freeze‐drying for 48 h. To ensure structural stability in aqueous environments, the freeze‐dried Uni‐CoF aerogels were subsequently subjected to thermal crosslinking at 120 °C for 3 h, inducing citric acid‐mediated esterification between the hydroxyl groups of the polysaccharides.

SHIFT was performed via a two‐step sequential protocol, with specific primary‐secondary combinations selected based on the target architecture as detailed in Table S1. For the fabrication of bi‐directional SHIFT aerogels, a 1.5 wt.% unidirectional agarose aerogel served as the primary template, infiltrated with varying secondary solutions, including 1.0 wt.% sodium alginate for mechanical studies, or diverse polymers such as CMC (2.5 wt.%), arabic gum (10.0 wt.%), gelatin (15.0 wt.%), CNC (2.0 wt.%), and PVA (5.0 wt.%) to demonstrate versatility. In all configurations, the primary aerogel was immersed in the corresponding secondary solution for 6 h under vacuum (Edwards RV8, Edwards Ltd., UK) to ensure complete degassing and infiltration. The sample was subsequently positioned on a cold finger tilted at a programmed angle (*θ* = 0°, 30°, 45°, 60°, or 90°) relative to the primary alignment axis and cooled with liquid nitrogen (‐196 °C) to establish a secondary directional temperature gradient. The frozen samples were then freeze‐dried for 48 h. Furthermore, to fabricate non‐agarose bi‐directional SHIFT (Bi‐SHIFT) aerogels, primary templates were additionally prepared using chitosan and alginate. Chitosan was dissolved in a 2% aqueous acetic acid solution to prepare a 2 wt.% chitosan solution, which was subsequently freeze‐cast. The frozen sample was then immersed in a 1.5 m aqueous NaOH solution at 4 °C for 6 h, where it slowly thawed and regenerated to form a water‐resistant chitosan gel. Similarly, a 2.5 wt.% aqueous alginate solution was freeze‐cast and subsequently crosslinked in a 2 wt.% aqueous CaCl_2_ solution at 4 °C for 6 h to yield a water‐resistant alginate gel. Thereafter, the prepared chitosan and alginate gels were washed with distilled water overnight to completely remove any residual NaOH and CaCl_2_. These gels were then freeze‐cast again and freeze‐dried for 48 h to obtain the final primary templates. These diverse polymer‐based primary aerogels were infiltrated with a 2 wt.% aqueous carboxymethyl cellulose (CMC) solution under vacuum for 6 h, followed by a secondary freeze‐casting step performed at an angle of 90°. Finally, the frozen samples were freeze‐dried for 48 h.

For the fabrication of radial‐SHIFT aerogels, a pure 5.0 wt.% radially freeze‐cast agarose aerogel was utilized as the primary template and infiltrated with a secondary solution of 2.0 wt.% CNC and 0.2 wt.% citric acid. Subsequently, the infiltrated scaffold was subjected to a sequential unidirectional freeze‐casting process to establish the final dual‐aligned architecture. Following freeze‐drying for 48 h, both the radial control and radial‐SHIFT aerogels containing CNC and citric acid were subjected to a thermal crosslinking step at 120 °C for 3 h to induce esterification‐mediated bonding.

To investigate the structural variation as a function of freezing temperature, the following procedures were conducted. First, to observe the morphological changes in the primary structure induced by freezing temperature, a 1.5 wt.% agarose hydrogel was freeze‐cast on a cold finger pre‐cooled to −80 °C and then freeze‐dried for 48 h. To observe the morphological changes in the secondary structure induced by freezing temperature, an agarose Uni‐FC scaffold (freeze‐cast at −196 °C) was infiltrated with a 1.0 wt.% alginate solution. The sample was then subjected to a secondary freeze‐casting process on a −80 °C cold finger, oriented orthogonally to the primary freezing direction, and freeze‐dried for 48 h to yield the final aerogel.

To analyze the relationship between the freezing rate and pore size as a function of distance from the cold finger, samples were prepared as follows. A 1.5 wt.% agarose hydrogel with dimensions of 2 cm × 2 cm × 6 cm (W × L × H) was placed on a cold finger pre‐cooled to −196 °C for unidirectional freeze‐casting. The frozen samples were then freeze‐dried for 48 h to yield aerogels, which were subsequently used to investigate the height‐dependent structural evolution.

### 4.3 Freezing Rate Analysis

The freezing rate of the gel during the directional freeze‐casting process was determined quantitatively via an image‐based tracking method. The gel sample was placed on a cold finger pre‐cooled to −196 °C to induce unidirectional freezing. During the process, the upward progression of the ice front within the gel was recorded, and its position was monitored at 1‐min intervals. The spatial coordinates of the ice front were extracted using Tracker software (Open Source Physics, USA), and the vertical displacement of the ice front was plotted as a function of time. To calculate the exact freezing rate, the region exhibiting a linear increase in height was first identified using a custom Python script. Subsequently, linear regression analysis was performed on this specific linear region using GraphPad Prism software (version 9.3, GraphPad Software, San Diego, CA, USA). The freezing rate was directly derived from the slope of the regression line, and the reliability of the fit was evaluated using the coefficient of determination.

### 4.4 Microstructural Characterization

The microstructural morphologies of the cuttlebone and fabricated aerogels were examined using a scanning electron microscope (SEM, EM30N, COXEM, Daejeon, Korea). Images were acquired at an accelerating voltage of 15 kV and a working distance of approximately 8 mm. Prior to imaging, the samples were coated with platinum for 40 s using an ion sputter coater (G10, COXEM) to prevent surface charging and ensure conductivity.

Confocal laser scanning microscopy (CLSM, K1‐Fluo, Nanoscope Systems, Daejeon, Korea) was employed to visualize the internal architectural features and infiltration behavior. For the structural characterization of the primary structures, the agarose precursor solution was stained with 0.001 wt.% Rhodamine B prior to gelation. Conversely, for the evaluation of infiltration kinetics during the immersion tests, the secondary sodium alginate solution was labeled with 0.001 wt.% Rhodamine B to trace the diffusion depth and distribution.

The three‐dimensional, non‐destructive microstructural morphology of the Bi‐SHIFT aerogels was examined using an X‐ray microscope (Xradia 620 Versa, Carl Zeiss, Oberkochen, Germany). The tomographic scans were performed at an accelerating voltage of 70 kV and a power of 8.5 W. A total of 2001 projections were acquired with an exposure time of 5 s per projection. The reconstructed 3D data were subsequently visualized and analyzed using Dragonfly 3D World software (Zeiss Edition, Comet, Montreal, QC, Canada).

### 4.5 Structural Analysis and Porosity Characterization

The bulk density (*ρ*) of the aerogels was determined gravimetrically using Equation (1):

(1) ρ=m/V

where *m* is the mass, and *V* is the volume of the aerogel. Subsequently, the porosity was calculated based on the ratio of the apparent density to the skeletal density, using Equation (2):

(2) $$\mathrm{Porosity}\;\left(\%\right)=\left(1\;-\;\frac{\rho_{\mathrm{aerogel}}}{\rho_{\mathrm{bulk}\;\mathrm{agarose}}}\right)\;\times \;100$$

where *ρ*_bulk agarose_​ is the density of solid agarose (approx. 0.55 g·cm^−^^3^).

The pore structure of the aerogels was quantified using the SEM images. The inter‐lamellar spacing, defined as the distance between parallel lamellar layers in the cross‐section, was measured using ImageJ software (version 1.52a, NIH, Bethesda, MD, USA). For statistical reliability, the average pore size was calculated based on measurements from 20 randomly selected locations per image.

### 4.6 Aqueous Stability and Capillary Force Modeling

To evaluate the water resistance and structural stability of the primary agarose, chitosan, and alginate aerogels, an immersion test in distilled water was conducted. The structural integrity of the samples was both qualitatively and quantitatively assessed across three distinct stages: the initial freeze‐dried state, the swollen state after 24 h of immersion, and the final re‐freeze‐dried state. Digital photographs were acquired at each stage to qualitatively monitor morphological changes. Furthermore, to quantitatively determine structural loss, the weight change ratio was calculated using Equation (3) by comparing the initial mass of the aerogels prior to immersion with their final mass obtained after the re‐freeze‐drying process:

(3) $$Weight\;change\;ratio=\frac{mass_{initial\;aerogel}}{mass_{after\;re-freeze-dried}}$$

To compare the transverse compressive stress–strain curves of chitosan and alginate with their respective capillary pressures, the capillary pressure of each material was calculated using the Young‐Laplace equation (4).

(4) $$P_{capillary}=\frac{2\gamma \cos\theta }{r}$$

where *γ* is the surface tension of water (72.0 mN·m^−^^1^), *θ* is a measured water contact angle with each polymer, and *r* is the average pore radius of each freeze‐cast polymer aerogel.

### 4.7 Evaluation of Infiltration Kinetics and Dimensional Stability

To optimize the sequential protocol, the infiltration kinetics and structural stability of the primary template were evaluated via a systematic immersion test. 1.5 wt.% unidirectional agarose aerogels were immersed in sodium alginate solutions with varying concentrations (0, 0.5, 1.0, and 2.0 wt.%) at 30 °C. To visualize the infiltration behavior, Rhodamine B was added to the alginate solutions as a fluorescent tracer. The samples were retrieved at predetermined time intervals (0.5, 1.0, 2.0, 3.0, 6.0, and 12.0 h) and immediately frozen at ‐80 °C, followed by freeze‐drying for 48 h.

To quantify the infiltration efficiency, the cross‐sections of the dried aerogels were imaged using a fluorescence microscope. The fluorescence intensity profiles were extracted across the normalized width of the aerogel cross‐section (ranging from −0.5 to 0.5). To evaluate the infiltration depth, the fluorescence difference (*Δ*FI) was calculated, focusing on the core region. Specifically, the mean fluorescence intensity within the central normalized distance range of −0.05 to 0.05 was determined for both the distilled water (DW) control (FI_DW_​) and the alginate‐infiltrated samples (FI_x%​_). The infiltration efficiency was calculated using Equation (5):

(5) $$\Delta FI=FI_{DW}-FI_{x\%}$$

Simultaneously, the dimensional stability was assessed by measuring the cross‐sectional area of the samples from the captured images. The degree of swelling or shrinkage was quantified by the area ratio using Equation (6):

(6) $$Area\;ratio=\frac{A_{t}}{A_{0}}$$

where *A*_0_​ is the initial cross‐sectional area of the primary aerogel, and *A*_t_​ is the area measured after an immersion time *t*.

### 4.8 Rheological Characterization of Secondary Polymer Solutions

The rheological behaviors of the secondary polymer solutions were evaluated using a digital rheometer (MCR 302e, Anton Paar, Graz, Austria) equipped with parallel plate geometries (diameter: 50 mm) and a gap setting of 0.5 mm. To assess the flow characteristics relevant to the infiltration process, the steady‐shear viscosity was measured as a function of shear rate over a range of 0.1 to 1000 s^−^^1^ at room temperature.

### 4.9 Surface Energy and Wetting Behavior Analysis

The surface wetting properties and surface energy components of the polymers were evaluated using a contact angle analyzer (DSA 100, KRÜSS GmbH, Hamburg, Germany). To prepare the solid substrates, polymer films were fabricated by casting 800 µL of each solution onto a glass slide, followed by drying under humid conditions at 35 °C for over 24 h.

For the measurements, sessile drops (9 µL) of two test liquids, deionized water (polar probe) and diiodomethane (dispersive probe), were dispensed onto the polymer film surfaces. The contact angle evolution was recorded for 20 s after the droplet made contact. The total surface energy (*γ*_s_​)​ was calculated by resolving it into dispersive (*γ* ^d^) and polar (*γ* ^p^​​) components using the Owens‐Wendt‐Rabel‐Kaelble (OWRK) method, based on Equation (7):

(7) $$\frac{\gamma_{L}\left(1+cos\theta \right)}{2\sqrt{\gamma_{L}^{d}}}=\sqrt{\gamma_{S}^{p}}\;\cdot \sqrt{\frac{\gamma_{L}^{p}}{\gamma_{L}^{d}}}+\sqrt{\gamma_{S}^{d}}$$

where ​​γ_*L*_, $\gamma_{L}^{d}$​, and $\gamma_{L}^{p}$​​ represent the total surface tension, dispersive component, and polar component of the test liquid, respectively, and *θ* is the measured contact angle.

To assess the interfacial compatibility between the primary scaffold and the secondary polymer solutions, the solid‐liquid interfacial tension (*γ*_SL_​) was determined using Equation (8).

(8) $$\gamma_{SL}=\gamma_{S}+\gamma_{L}-2\left(\sqrt{\gamma_{S}^{d}\cdot \gamma_{L}^{d}}+\sqrt{\gamma_{S}^{p}\cdot \gamma_{L}^{p}}\right)$$

Subsequently, the work of adhesion (*W*_a_​), which quantifies the interaction strength at the interface, was calculated using Equation (9).

(9) $$W_{ad}=\gamma_{S}+\gamma_{L}-\;\gamma_{SL}=2\left(\sqrt{\gamma_{S}^{d}\cdot \gamma_{L}^{d}}+\sqrt{\gamma_{S}^{p}\cdot \gamma_{L}^{p}}\right)$$

High values of *W*_ad_​ and low values of *γ*_SL_​ indicate favorable wetting and strong adhesion between the materials.

### 4.10 Mechanical Characterization and Anisotropy Evaluation

The mechanical performance of the aerogels was comprehensively evaluated through uniaxial compression, cyclic compression, and single‐edge notched bending (SENB) test. All measurements were conducted using a universal testing machine (UTM, GB/LRX Plus, Lloyd, West Sussex, UK) equipped with a 500 N load cell under controlled environmental conditions of 25 °C and 45% relative humidity.

For the uniaxial compression tests, cuboid specimens (dimensions: 15 mm × 10 mm × 10 mm) were prepared. For mechanical characterization, specimens were prepared at approximately 10 mm scale to ensure reproducible geometry, minimize position‐dependent structural heterogeneity, and allow consistent comparison among Non‐freeze casting, Uni‐FC, Uni‐CoF, and Bi‐SHIFT samples. To investigate structural anisotropy, the compressive load was applied either parallel or perpendicular to the primary freezing axis. The tests were performed at a strain rate of 1 mm·min^−^^1^ up to a strain of 90% or until fracture occurred. The compressive modulus was calculated from the slope of the linear elastic region within the 0% to 1% strain range. The toughness was quantified as the total energy absorbed per unit volume, calculated by integrating the area under the stress–strain curve up to a strain of 10%. The specific compressive modulus and specific energy absorption were then determined by dividing the modulus and toughness by the density of the respective material. The anisotropic ratio was calculated as the compressive modulus measured parallel to the primary structure to that measured perpendicular to it, using Equation (10).

(10) $$Anisotropic\;ratio=\frac{E_{∥}}{E_{⊥}}$$

Cyclic compression tests were conducted to assess the fatigue resistance and shape recovery properties relative to the microstructural alignment. Cuboid specimens (15 mm × 10 mm × 10 mm) were prepared with the secondary alignment axis oriented at varying angles (*θ* = 0°, 30°, 45°, 60°, and 90°) with respect to the primary alignment axis. The compressive load was applied perpendicular to the primary freezing axis. The samples were subjected to 50 loading‐unloading cycles at a strain rate of 5 mm·min^−^^1^ with a maximum strain of 10%. The stress recovery ratio was calculated by comparing the maximum stress at the 50^th^ cycle with that of the first cycle to quantify cyclic mechanical durability. For the long‐term cyclic compression test, the samples were subjected to 500 loading‐unloading cycles at a strain rate of 5 mm·min^−^^1^ with a maximum strain of 10%. The stress recovery ratio was calculated as the ratio of the maximum stress at the 500^th^ cycle to that at the first cycle to quantify cyclic mechanical durability.

Fracture toughness was evaluated by the SENB test using the three‐point bending method. Rectangular specimens (dimensions: 15 mm × 10 mm × 5 mm) were prepared with a prefabricated notch (length: 1 mm) positioned at the center of the bottom edge. The tests were performed in a three‐point bending configuration with a support span of 14 mm at a displacement rate of 1 mm·min^−^^1^. The fracture energy was determined from the load‐displacement curves to analyze the crack propagation resistance provided by the architecture.

### 4.11 Quantitative Analysis of Microstructural Alignment

The degree of structural anisotropy and the preferential alignment of the polymer lamellae were quantitatively evaluated using the Directionality plugin within the FIJI distribution of ImageJ software. This computational tool was employed to generate orientation histograms by analyzing the local gradient vectors of the microstructural features in the aerogel cross‐sections. The polymer alignment angles were quantified across a range of 0° to 90° relative to the principal axis. To visualize the distribution of fiber orientations, the resulting data were plotted as polar diagrams (circular bar graphs) using Microsoft Excel (version 2024, Microsoft, Redmond, WA, USA).

### 4.12 Analysis of Fluid Transport and Wicking Kinetics

Fluid transport behaviors within the porous architectures were investigated using two distinct protocols: qualitative visualization of flow patterns and quantitative analysis of wicking kinetics. For all experiments, a 0.1% (w/v) aqueous red dye solution was employed as a tracer to track the fluid movement.

To visualize the multidirectional fluid transport, radially freeze‐cast aerogels and radial‐SHIFT aerogels were examined. A total volume of 200 µL of the dye solution was supplied to the basal surface of the aerogel at a controlled flow rate of 0.1 mL·min^−^^1^ using a syringe pump. The fluid permeation process was recorded as a video sequence at 30 frames per second (fps). Representative snapshots were subsequently extracted from the footage to visualize the radial propagation of the fluid.

For the quantitative evaluation of anisotropic wicking behavior, unidirectionally freeze‐cast aerogels and bi‐directional SHIFT aerogels were tested. A 400 µL droplet of the dye solution was dispensed onto a hydrophobic polytetrafluoroethylene (PTFE) film. One corner of the rectangular aerogel specimen was brought into contact with the droplet to initiate capillary wicking. The fluid uptake was recorded at 30 fps, and frames were extracted at 10‐frame intervals using video processing software (Clipchamp, version 4.4.10420.0, Microsoft). The wicking propagation was quantified by measuring the lateral absorption distance (*d*_lat_​), vertical absorption distance (*d*_vert_​) using ImageJ.

### 4.13 Statistical Information

All quantitative data are presented as the mean ± standard deviation (SD) derived from at least three independent experiments (n ≥ 3), unless otherwise specified in the figure captions. Statistical analyses and graphical plotting were performed using GraphPad Prism software and Microsoft Excel. Differences between two groups were evaluated using an independent Student's t‐test, whereas comparisons among multiple groups were performed using a one‐way analysis of variance (ANOVA) followed by Tukey's post hoc test. Statistical significance was defined at a probability value (P) of less than 0.05.

## Author Contributions

**K.S**. Methodology, Software, Validation, Formal Analysis, Investigation, Data Curation, Writing – Original Draft, Writing – Review & Editing, Visualization. **S.S**. Conceptualization, Writing – Original Draft, Writing – Review & Editing, Visualization, Supervision, Project Administration, Funding Acquisition.

## Conflicts of Interest

The authors declare no conflicts of interest.

## Supporting information




**Supporting File 1**: advs76283‐sup‐0001‐SuppMat.docx.

**Supporting File 2**: advs76283‐sup‐0002‐MovieS1.mp4.

**Supporting File 3**: advs76283‐sup‐0003‐MovieS2.mp4.

**Supporting File 4**: advs76283‐sup‐0004‐MovieS3.mp4.

## Data Availability

The data that support the findings of this study are available from the corresponding author upon reasonable request.
