# Supplementary material for: Programmable Multi‐Axially Aligned Aerogels via Sequential Freeze‐Casting for Tailored Anisotropy and Tunable Mechanics
Source: Adv Sci (Weinh). 2026 Jun 23;13(48):e76283. doi: 10.1002/advs.76283 (PMC13336433; doi:10.1002/advs.76283)
Supplement: Supplementary file 1 [file ADVS-13-e76283-s001.docx]

Supporting Information

**Programmable Multi-Axially Aligned Aerogels via Sequential Freeze-Casting for Tailored Anisotropy and Tunable Mechanics**

*Kiho Sung*^1^ *& Sungchul Shin*^1,2,*^

^1^Department of Agriculture, Forestry, and Bioresources, Seoul National University, Seoul, Republic of Korea

^2^Research Institute of Agriculture and Life Sciences, Seoul National University, Seoul, Republic of Korea
Corresponding author: sungssc@snu.ac.kr

**This file includes:**

Figure S1 to S22

Table S1

Supporting information reference

**Other supplementary materials for this manuscript include the following:**

Supplementary Movie 1 to 3

**Figure S1. Structural characterization of primary agarose aerogels.** (a) Porosity of primary aerogels as a function of agarose concentration. Bars represent mean values, and individual data points are overlaid as grey dots. Data are presented as mean ± SD (n = 3). Statistical comparison was performed using one-way ANOVA followed by Tukey’s post hoc test (****P < 0.0001). (b) Pore size distribution of primary aerogels prepared with varying agarose concentrations. Data are presented as a violin plot (n = 60).

**Figure S2. Effect of cold-source condition on primary and secondary microstructures in freeze-cast agarose/alginate aerogels.** Cross-sectional SEM images of primary agarose scaffolds freeze-cast under cold-source conditions of (a) -80 °C and (b) liquid nitrogen cooling. (c) Inter-wall spacing of the primary agarose scaffolds formed under different cold-source conditions. Data are presented as a violin plot (n > 60). Statistical comparison in (c) was performed using Student’s t-test (***P < 0.001). Cross-sectional SEM images of agarose/alginate SHIFT aerogels with secondary alginate structures formed under cold-source conditions of (d) -80 °C and (e) liquid nitrogen cooling. (f) Inter-wall spacing of the secondary alginate structures formed under different cold-source conditions. Data are presented as a violin plot (n > 60). Statistical comparison in (f) was performed using Student’s t-test (****P < 0.0001).

**Figure S3. Hydrolytic stability of primary agarose aerogels.** Weight change ratio of primary aerogels with varying agarose concentrations after immersion in distilled water for 24 h. Data are presented as mean ± SD (n = 6). Statistical comparison was performed using one-way ANOVA followed by Tukey’s post hoc test (*P < 0.05).

**Figure S4. Structural stability of primary agarose aerogels during swelling and re-drying cycles.** (a) Optical photographs of as-prepared primary aerogels with varying agarose concentrations (0.5-5.0 wt%). (b) Swollen aerogels after immersion in distilled water for 24 h. (c) Re-dried aerogels obtained after re-freezing and subsequent freeze-drying.

**Figure S5. Transverse compressive properties of primary agarose aerogels.** (a) Schematic illustration of the transverse compression test for unidirectional freeze-cast (Uni-FC) primary agarose aerogels with varying concentrations (0.5 to 5.0 wt%). (b) Compressive stress-strain curves of primary agarose aerogels prepared with varying concentrations. The boxed region highlights the low-strain regime detailed in Fig. 2c. (c) Compressive modulus of primary agarose aerogels as a function of agarose concentration. Data are presented as mean ± SD (n = 3).

**Figure S6. Swelling behavior and structural stability of primary Uni-FC agarose aerogels.** (a) Optical photographs (top) and corresponding CLSM microstructures (bottom) of the primary unidirectional freeze-cast aerogel (1.5 wt%) before and after immersion in distilled water for 24 h. (b) Normalized volume of primary aerogels (1.5 wt%) after immersion in distilled water for 0, 1, and 24 h. Data are presented as mean ± SD (n = 3). Statistical comparison was performed using one-way ANOVA (ns, not significant).

**Figure S7. Structural integrity of the primary agarose template under multi-step freeze-casting cycles.** CLSM images display the morphology after (a) single, (b) double, and (c) quadruple freeze-casting cycles. The 1.5 wt% agarose template exhibits sufficient mechanical stability to withstand multiple re-freezing sequences without degradation of the primary alignment (green arrow). Colored arrows indicate the direction of the temperature gradient for each step.

**Figure S8. Rheological characterization of secondary alginate solutions.** (a) Steady-shear viscosity as a function of shear rate for alginate solutions with varying concentrations (0.5, 1.0, and 2.0 wt%). (b) Zero-shear viscosity (η_0_) as a function of concentration, derived from the Cross model fitting in (a). Data are presented as mean ± SD (n = 3).

**Figure S9. Spatio-temporal infiltration kinetics of secondary alginate solutions.** Fluorescence intensity profiles measured across the normalized cross-section (-0.5 to 0.5) of primary aerogels. (a) Control profiles of primary aerogels immersed in distilled water (DW) containing 0.001 wt% Rhodamine B tracer over time (0.5 to 12.0 h). (b) Schematic definition of ΔFI, calculated as the intensity difference at the core between the DW control and alginate-infiltrated samples (x wt%). (c-h) Comparative intensity profiles of alginate solutions with varying concentrations (0.5, 1.0, and 2.0 wt%) after immersion times of (c) 0.5 h, (d) 1.0 h, (e) 2.0 h, (f) 3.0 h, (g) 6.0 h, and (h) 12.0 h.

**Figure S10. Rheological characterization of diverse secondary polymer candidates.** Steady-shear viscosity is plotted as a function of shear rate for solutions with varying concentrations of (a) carboxymethyl cellulose (CMC), (b) arabic gum, (c) fish gelatin, (d) cellulose nanocrystal (CNC), and (e) polyvinyl alcohol (PVA).

**Figure S11. Structural and mechanical stability of non-agarose primary templates for SHIFT.** (a) Optical images of regenerated chitosan and Ca²⁺-crosslinked alginate aerogels after initial lyophilization, rehydration, and re-lyophilization. (b) Mass retention of chitosan and alginate aerogels after rehydration. Data are presented as mean ± SD (n = 6). (c) Cross-sectional SEM images of freeze-cast chitosan and alginate aerogels showing aligned porous architectures. (d) Corresponding pore sizes of the chitosan and alginate aerogels. Data are presented as mean ± SD (n = 20). Transverse compressive stress-strain curves of (e) chitosan and (f) alginate aerogels. The red dashed lines indicate the estimated capillary pressures calculated from the contact angle and pore size of each material. (g) Compressive strain at the estimated capillary pressure. Both non-agarose primary templates retained structural integrity under aqueous rehydration and exhibited sufficient mechanical stability against capillary-pressure-driven deformation. Data are presented as mean ± SD (n = 3).

**Figure S12. SHIFT aerogels fabricated using non-agarose primary templates.** Cross-sectional SEM images of Bi-SHIFT aerogels fabricated using (a) regenerated chitosan and (b) Ca²⁺-crosslinked alginate as primary scaffolds, followed by infiltration and secondary freeze-casting of CMC. Both non-agarose primary scaffolds supported the formation of secondary aligned CMC structures within the preformed porous network, demonstrating that SHIFT is not limited to agarose-based primary templates.

**Figure S13. Surface wetting characterization of polymer films for OWRK analysis.** (a) Representative optical images of water droplets on various polymer films. (b) Measured contact angles of water. (c) Representative optical images of diiodomethane (DIM) droplets on polymer films. (d) Measured contact angles of DIM. In (b) and (d), data are presented as mean ± SD (n = 6).

**Figure S14. Internal microstructures of the (a) Uni-FC, (b) Uni-CoF, and (c) Bi-SHIFT (90°) aerogels.** Uni-FC exhibits a unidirectionally aligned porous structure formed from agarose. Uni-CoF, prepared by single-step co-freezing of agarose and alginate, also shows an unidirectionally aligned architecture without orthogonal secondary walls. Bi-SHIFT (90°) displays a hierarchically integrated architecture in which secondary structures bridge the primary aligned channels along an orthogonal axis.

**Figure S15. Mechanical comparison between isotropic control and anisotropic freeze-cast aerogels.** (a) Schematic illustration of the compression test for the isotropic (non-freeze-cast) aerogel. (b) Representative compressive stress-strain curve of the isotropic aerogel. (c, d) Comparison of compressive modulus between the isotropic control and three freeze-cast variants (Uni-FC, Uni-CoF, and Bi-SHIFT) measured in the (c) longitudinal (axial) and (d) transverse directions. Data are presented as mean ± SD (n = 3), with individual measurements overlaid as grey dots.

**Figure S16. Ashby plots comparing mechanical anisotropy and specific compressive modulus among representative freeze-cast systems.** Ashby plots correlating the anisotropy ratio with the specific compressive modulus in the (a) longitudinal (axial) and (b) transverse directions for the present aerogels and representative freeze-cast systems reported in the literature. The anisotropy ratio is defined as the ratio of longitudinal (axial) modulus to transverse modulus (E∥/E⊥). Reference categories: natural polymers (main text reference: [43-45], supporting information reference [1, 2]), synthetic polymers (main text reference: [48], supporting information reference [3, 4]), and ceramics (main text reference: [21, 46]).

F**igure S17. Mechanical properties of aerogels under transverse compression.** (a) Energy absorption capacity calculated in the low-strain regime. (b) Compressive modulus derived from the initial linear elastic region. The samples include agarose (Uni-FC), agarose/CNC (Bi-SHIFT) with low interfacial affinity, and agarose/alginate (Bi-SHIFT) with high interfacial affinity. Data are presented as mean ± SD (n = 3). Statistical comparisons were performed using one-way ANOVA followed by Tukey’s post hoc test (ns, not significant; *P < 0.05; **P < 0.01).

**Figure S18.** **Programmable angular architectures of Bi-SHIFT aerogels.** CLSM micrographs exhibiting microstructures engineered with varying secondary freezing angles (θ = 0°, 30°, 45°, 60°, and 90°). The angle θ is defined as the relative inclination between the primary alignment axis and the secondary temperature gradient.

**Figure S19. Long-term cyclic stability of SHIFT aerogels with different secondary alignment angles.** (a) Evolution of normalized compressive stress during 500 cycles of repeated compression. (b) Stress recovery ratio after 500 cycles of repeated compression. Data are presented as mean ± SD (n = 3). Statistical comparison in (b) was performed using one-way ANOVA followed by Tukey’s post hoc test (*P < 0.05).

**Figure S20. SEM analysis of SHIFT aerogels before and after 50 cycles of repeated compression.** The top and bottom rows display the microstructures of the aerogels before compression and after 50 cycles of repeated compression followed by unloading, respectively. From left to right, the columns correspond to Bi-SHIFT architectures with secondary alignment angles of 0°, 45°, and 90°. After 50 cycles, the 0° architecture exhibits reduced spacing between adjacent aligned layers and local bending deformation. The 90° architecture shows buckling and local fracture of vertically oriented walls. In contrast, the 45° architecture largely preserves its inclined secondary structures without pronounced fracture, indicating improved structural integrity under repeated compression.

**Figure S21. Wet-state mechanical evaluation of Radial-SHIFT aerogels after water-flow testing.** (a) Representative compressive stress-strain curves of Radial-SHIFT aerogels measured in the dry state before fluid transport and in the wet state after fluid transport. The wet sample exhibited a reduced compressive response compared with the dry sample. (b) Compressive modulus of Radial-SHIFT aerogels before and after fluid transport, showing a substantial decrease in modulus under the post-transport wet condition. Data are presented as mean ± SD (n = 3).

**Figure S22. Assessment of the accessible freezing distance in the cold finger-based freeze-casting setup.** (a) Photograph of a large-area primary freeze-cast agarose template, showing that the lateral dimension can be expanded by increasing the mold and copper plate area in a single freeze-casting step. (b) Time-dependent freezing-front propagation during directional freeze-casting of a height-extended agarose sample. (c) Height-dependent pore-size distribution of the primary scaffold. Relatively uniform pore sizes were maintained up to approximately 4.5 cm from the cold source, whereas the distribution broadened at greater distances. Data are presented as violin plots (n > 70). Statistical comparisons were performed using one-way ANOVA followed by Tukey’s post hoc test (ns, not significant). (d) Representative cross-sectional SEM images of primary scaffolds collected at heights of 1.5, 3.5, and 5.5 cm from the cold source.

**Table S1. Summary of fabrication protocols and material compositions.** Detailed list of freezing methods, specific concentrations for primary and secondary polymer precursors, and corresponding figure references.

References

[1]	B. Chen, Q. Zheng, J. Zhu, J. Li, Z. Cai, L. Chen, S. Gong, *RSC advances*, *6* (99), 96518 (2016).

[2]	X. Zhang, M. Liu, H. Wang, N. Yan, Z. Cai, Y. Yu, *Carbohydrate polymers*, *208*, 232 (2019).

[3]	L. Huang, J. Hua, Y. Yang, *Polymer Composites*, *46*, S413 (2025).

[4]	S. Fushimi, T. Nagakura, A. Yonezu, *Polymer Testing*, *63*, 605 (2017).
